# Nucleic acid probe-functionalized self-powered sensor for detection of biomarkers in clinical urine samples

**DOI:** 10.1126/sciadv.aeg5086

**Published:** 2026-08-05

**Authors:** Qitao Zhou, Along Gao, Xiali Yang, Huageng Liang, Jing Pan, Shaoguang Li, Hui Li, Caofeng Pan, Fan Xia

**Affiliations:** ^1^ State Key Laboratory of Geomicrobiology and Environmental Changes, Faculty of Materials Science and Chemistry, China University of Geosciences, Wuhan 430074, China.; ^2^ Union Hospital, Tongji Medical College, Huazhong University of Science and Technology, Wuhan 430030, China.; ^3^ Institute of Atomic Manufacturing, Beihang University, Beijing, 100191 China.

## Abstract

Triboelectric nanogenerator (TENG)-based self-powered biosensors can reduce ecological risks from energy storage device disposal and complex electronics manufacturing, offering cost-effective and eco-friendly diagnostic methods. However, in the intricate charge environment of urine, existing devices are difficult to specifically capture targets and produce recognizable changes in surface charge and the resulting electrical signals, leading to insufficient detection specificity and sensitivity. Herein, for the first time, the problem is addressed by a TENG-based self-powered sensor for clinical urine detection. Self-charged nucleic acid probe modification and targeted conformational change induce specific surface charge variation; regulating system ionic strength matches Debye length with probe conformational scale to maximize this variation. A double-electrode device efficiently converts surface charge changes into distinct output signals, boosting sensing performance in intricate charged environments. This sensor achieves sensitive detection of ATP (269 pM) and miR-21 (328 pM) in artificial urine, effectively monitors urinary ATP elevation in cystitis patients, showing potential for low-cost, easy-to-operate home health monitoring, promoting health equity.

## Teaser

A self-powered sensor based on a triboelectric nanogenerator is developed to achieve clinical urine detection.

## INTRODUCTION

Portable health monitoring devices are gaining increasing significance. These devices do not rely on high-precision, expensive, and energy-intensive medical equipment, nor do they require specialized personnel (*1*). For example, through non-invasive testing of urine rich in biochemical markers, these devices can provide a wealth of health monitoring information (*2*–*6*). However, the widespread adoption of such devices may conflict with the Sustainable Development Goals set by the United Nations. Firstly, when the batteries powering these devices are discarded and not properly managed, they can have a severe impact on the environment. Moreover, these devices rely on energy-intensive manufacturing processes, critical metals, and fossil-based plastics, posing significant risks in terms of carbon emissions, ecological toxicity, and electronic waste (*7*). Additionally, the current cost of such devices is generally high (*8*, *9*), preventing people in resource-constrained regions from enjoying their convenience (*10*). Addressing the aforementioned challenges, the self-powered active biochemical sensors based on triboelectric nanogenerators (TENGs) have the potential to offer cost-effective and eco-friendly diagnostic methods (*11*–*14*). This is attributed to their independence from external power supplies, low cost, and minimal potential pollution arising from their simple structure, thereby contributing to the promotion of health equity and sustainable social development.

Unfortunately, to date, even the most advanced self-powered active biochemical sensors based on solid-liquid interface TENGs can only detect relatively simple solution systems and cannot achieve in-situ detection of complex urine samples (*15*, *16*). This is mainly because urine contains high concentrations of salts and various charged substances, which form an intricate charge environment. Self-powered sensor based on solid-liquid interface TENG rely on the changes in the surface charge state of triboelectric materials induced by target molecules, which further alter the output electrical signal intensity for detection. However, this working mechanism is difficult to operate effectively in such an intricate charge environment. Firstly, most existing self-powered sensors directly adopt traditional triboelectric materials without specific modification for particular detection targets. Their surface charges are easily affected by interferents in urine, leading to severely disturbed output electrical signals and insufficient detection specificity (*17*–*19*). Secondly, the intricate charge environment of urine reduces the surface charge density of triboelectric materials through the formation of electrical double layers and electrostatic neutralization effects (*20*, *21*). As a result, the slight changes in surface charge caused by target binding cannot be converted into distinguishable output signal differences, resulting in low device sensitivity. Therefore, to realize accurate detection of complex liquid samples such as urine, TENG-based self-powered sensors urgently need breakthroughs in both specificity and sensitivity.

In this work, for the first time, we introduce a self-powered sensor that can realize detection of biomarkers in the complex liquid sample of urine. By modification of the nucleic acid probe, even in the context of the intricate charged environment of urine, the conformational alteration of the self-charged nucleic acid probe before and after capturing the target can cause specific changes in the charged properties of the triboelectric material surface. Subsequently, the device with double-electrodes structure ensures that even small changes in surface charge caused by alteration in nucleic acid probe conformation can be amplified as larger variation in the output electrical signal. Through the successful detection of two important disease-related urinary biomarkers, adenosine triphosphate (ATP) and miR-21 (*22*, *23*), as well as a series of characterizations, the sensing mechanism and the improved sensitivity and specificity of the device are fully verified. Additionally, by studying the impact of Debye screening on detection performance, optimization of the detection performance is achieved. Ultimately, this device achieves the detection of abnormally elevated ATP levels in urine samples from cystitis patients, successfully distinguishing cystitis patients from healthy individuals.

## RESULTS

### Design and operating principle of self-powered urine sensor

**Fig. 1.**
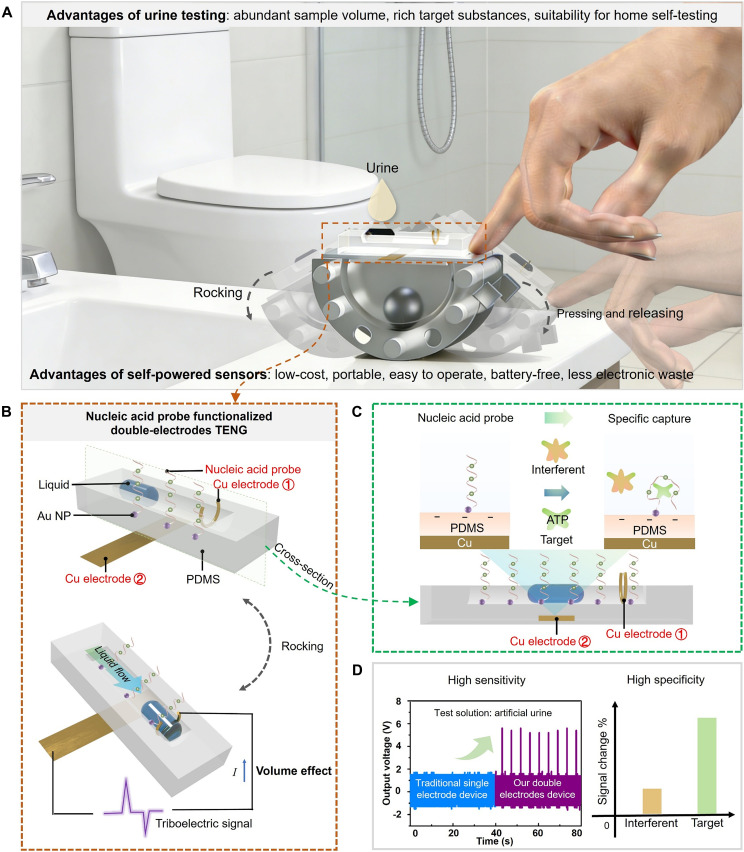
Schematic of self-powered urine sensor. (**A**) Advantages of urine testing and self-powered sensors, along with a schematic illustration of the self-powered sensor developed in this paper. (**B**) The schematic diagram of the nucleic acid probe-functionalized double-electrodes TENG-based self-powered sensor. (**C**) Schematic diagram of target-specific capture achieved by nucleic acid probes on the surface of triboelectric materials. (**D**) The device developed in this paper achieves an improvement in both sensitivity and specificity for complex liquid samples.

In order to more effectively utilize surface charges and acquire stronger electrical signals, the fluidic device in this study adopt the double-electrodes architecture (fig. S1) from our previous work (*14*) to introduce volume effects (*24*) (Fig. 1B). Briefly, when the liquid comes into contact with the top electrode and bridges it to the negatively charged polydimethylsiloxane (PDMS) surface, electrostatic induction effect occurs. This induces electrons to spontaneously flow from top electrode to the bottom electrode, thereby generating an electrical signal output between them (fig. S2). From Fig. 1D and movie S1, it can be seen that, when single electrode device is used to detect artificial urine, there is almost no obvious output voltage signal. Because single electrode device can only utilize extremely limited surface charges (fig. S3). After connecting the double electrodes, the device changes from utilizing the interfacial effect to the more sufficient volume effect (*25*) of system charge utilization, so the output voltage reaches nearly 6 V, while achieving a maximum instantaneous power density output of 12.8 W m^−3^ (fig. S4).

Subsequently, in order to achieve specific capture of two important biomarkers, ATP and miR-21 in urine, corresponding nucleic acid probes are modified onto the triboelectric material surface. Firstly, Au nanoparticles (Au NPs) are prepared using the sodium citrate reduction method and assembled onto PDMS surface (*26*). The SEM image (fig. S5) and UV-visible spectrophotometry (fig. S6) indicate that Au NPs with a main particle size distribution around 60 nm (fig. S7) were successfully modified on the surface. Then, nucleic acid probes targeting ATP and miR-21 are grafted onto the Au NPs via Au-S bonds to obtain a sensing surface with capturing functionality for the biomarkers (fig. S8). Nucleic acid probes are chosen to enhance device specificity for the following reasons: (i) They are natural biomolecules with strong specific recognition capabilities for molecular targets (*27*, *28*); (ii) The established design and screening techniques for different biomarkers make nucleic acid probes universally applicable and specific; (iii) The last but the most important reason is that the nucleic acid probe itself carries negative charges, which can bring changes in the distribution of charges on the surface of triboelectric material corresponding to the conformation change of nucleic acid probe before and after capturing the target (Fig. 1C). Considering that the device has an extremely simple structure, and the materials used are either low-cost or required in minimal quantities, the overall cost of the device is less than 0.6 US dollar.

All biological samples contain high concentrations of mobile ions, as a consequence, the electrostatic neutralization effect and screening effect (Debye screening effect) from these ions can greatly attenuate biosensor signals and reduce the impact of conformational changes in nucleic acid probes on the output signal. This fundamental physical effect, which manifests as the electrical double layer, prevents many electronic detection platforms from becoming broadly useful (*29*).

In the early stage, to avoid the intricate charged environment arising from high concentrations of mobile ions and charged substances, the devices are washed and dried after respective treatments, followed by addition of an equal volume of 0.001× PBS. Then, the output electrical signals are generated by uniformly shaking the devices. In 0.001× PBS, the Debye length (λ_D_ = 23.7 nm) is much greater than the length of the nucleic acid probe. Thus, the charge on the nucleic acid probe will not be affected by the screening effect. The output electrical signals can illustrate changes in the surface charge state of sensing surfaces before and after surface modifications, as well as after further capturing the target. In other words, the 0.001× PBS acts as a fixed positive triboelectric material.

**Fig. 2.**
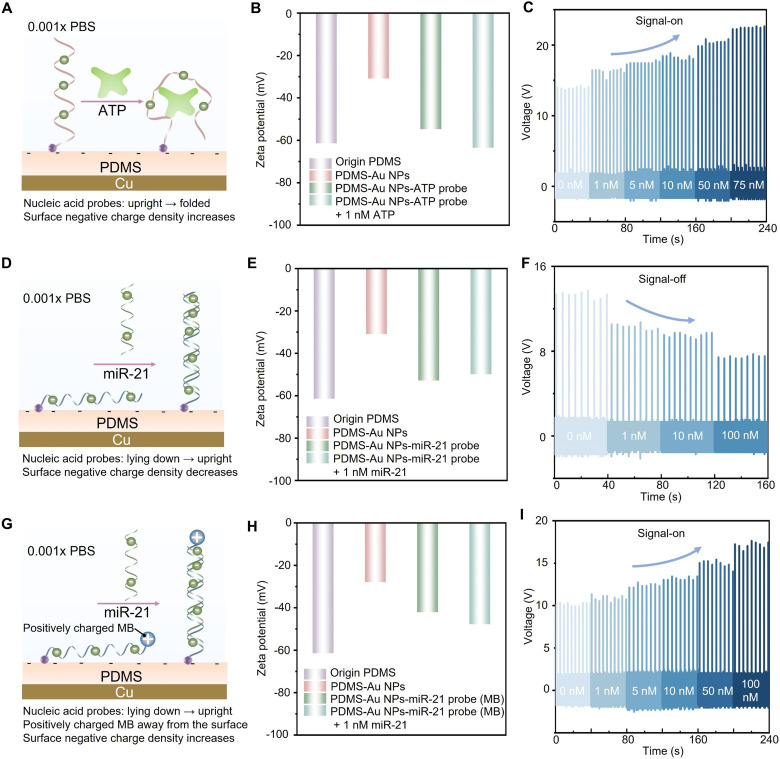
Working principles and output performance characterization of self-powered sensors for different biomarkers. (**A**) Schematics of the sensor modified with ATP aptamer used to detect ATP. (**B**) Zeta potentials of the as-prepared PDMS surface and PDMS surface after different treatments. (**C**) Output voltages of the sensor modified with ATP aptamer, as a function of ATP concentration. (**D**) Schematics of the sensor modified with nucleic acid probes used to detect miR-21. (**E**) Zeta potentials of the as-prepared origin PDMS surface and PDMS surface after different treatments. (**F**) Output voltages of the sensor modified with nucleic acid probes used to detect miR-21, as a function of miR-21 concentration. (**G**) Schematics of the sensor modified with nucleic acid probe (modified with MB) used to detect miR-21. (**H**) Zeta potentials of the as-prepared PDMS surface and PDMS surface after different treatments. (**I**) Output voltages of the sensor modified with nucleic acid probe (modified with MB), as a function of miR-21 concentration. All output voltage signals are measured after the device has undergone different treatments, followed by cleaning the device, then adding an equal amount of 0.001× PBS to the device, and shaking the device at the same frequency.

Firstly, ATP, a relatively simple and important small molecule biomarker, has been detected. As shown in Fig. 2A, a nucleic acid probe with a short chain length is chosen as the probe. Then the change of output performance after each operation step of the biochemical sensor is investigated (Fig. 2C). Due to the short length of the ATP probe, there is a change from an upright to a folded state before and after capturing ATP. This process brings the negatively charged nucleic acid backbone closer to the negatively charged PDMS surface (Fig. 2A), similar to creating a new surface with a higher negative charge density, thereby enhancing the output electrical signal. The zeta potential characterization of different surfaces validates the above process (Fig. 2B). Contact angle measurements were performed on the triboelectric material surface at each step of the preparation process as well as before and after target capture. It can be observed that the trend of contact angle variation is similar to that of zeta potential (fig. S9). Expectedly, the output electrical signal of the device continuously strengthens as the ATP concentration increases. When the ATP concentration reaches 75 nM, the output voltage increases to 22.2 V (Fig. 2C). The corresponding output current and transferred charge of device also follows similar trend (fig. S10, A and B). However, when ATP is detected using either a device without modification nucleic acid probe targeting ATP (fig. S10C) or a device modified the nucleic acid probe targeting miR-21 (fig. S10D), no specific enhancement in the output electrical signal is observed. This result confirms that the modification of nucleic acid probes targeting ATP indeed achieves specificity in detection.

Next, we conduct a test on another larger and important biomarker molecule, miR-21. First, following the conventional approach, the complementary strand of miR-21 is chosen as specific probe for miR-21 recognition (Fig. 2D). Then the change of output performance after each operation step of the biochemical sensor is investigated (fig. S11). Due to the longer length of the nucleic acid probe targeting miR-21, it initially lies in a fallen-over state before capturing miR-21. The negative charges on the nucleic acid backbone increase the negative charge density of the triboelectric material surface. However, after capturing miR-21, the double-stranded structure becomes rigid and stands upright, increasing the distance between the negative charges on the nucleic acid backbone and the triboelectric material surface, which is akin to reducing the negative charge density on the triboelectric material surface. Therefore, the output voltage exhibits an enhancement after modifying the probe but decreases after capturing the target. To corroborate the above explanation, the zeta potential of the triboelectric material surfaces at each stage are measured, revealing that its variation aligns with the previously mentioned trend in the intensity of the output electrical signals (Fig. 2E). Corresponding to the sensing mechanism, the device exhibits a trend where the intensity of the output voltage decreases as the target concentration increases (Fig. 2F).

However, the afore mentioned signal-off detection strategy is not the optimal solution when high concentrations of salt ions in urine weaken the output electrical signal. In comparison, developing a signal-on detection strategy is more favorable for urine testing. Here, we modify the positively charged methylene blue (MB) molecule (*30*) at top end of the nucleic acid probe for miR-21. Surprisingly, the peak values of output voltage display an upward trend from 10.3 V to 17.4 V with increasing concentration of miR-21 from 0 nM to 100 nM (Fig. 2I). Similarly, the corresponding short-circuit current and transferred charge also demonstrates similar trends (fig. S12).

This is due to the fact that, before capturing miR-21, the probe is initially in a fallen-over state, and the positively charged MB molecule weaken the negative charge on the triboelectric material surface. However, after capturing miR-21, the probe stands upright, increasing the distance between MB and the triboelectric material surface (Fig. 2G), which significantly reduces its weakening effect on the negative charge. Additionally, the total number of negative charges on the nucleic acid backbone increases after capturing miR-21. Ultimately, due to the competition among various factors, the output electrical signal of the device is stronger after capturing the target than before, successfully achieving the construction of a signal-on detection strategy. The zeta potential at each stage further confirms this result (Fig. 2H). Moreover, the device’s selectivity is examined by testing miR-21 with the bare PDMS device and mismatch target RNA with the device modified miR-21-probe-MB. As shown in fig. S13, A and B, the output signals of these devices do not show an enhancement after contacting the target in the above cases. The above results indicate that the nucleic acid probes conformational alterations following the capture of targets regulate the charged state of the triboelectric material surface. This change in charged state is then converted into a significant variation in output electrical signals through the double-electrodes device. The combination of these two steps achieves an improvement in both specificity and sensitivity for the self-powered device.

**Fig. 3.**
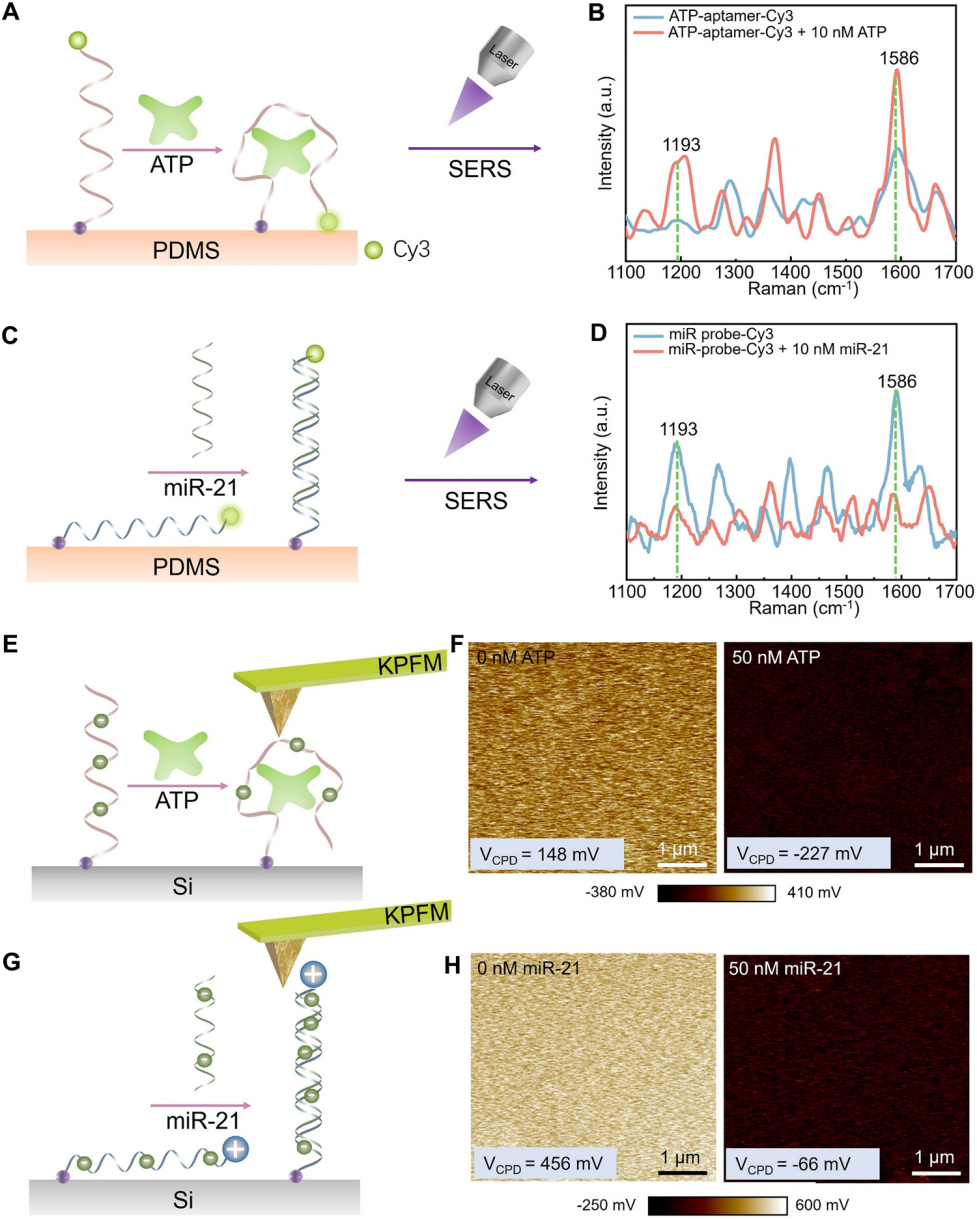
Characterization of conformational changes and charged state variations on triboelectric surfaces pre/post target capture by nucleic acid probes. (**A**) Schematic illustration of the conformational shifts of Cy3-labeled probes on Au NP/PDMS before/after ATP capture. (**B**) SERS spectra of Cy3 before/after ATP capture. (**C**) Schematic illustration of the conformational changes for miR-21 capture. (**D**) SERS spectra of Cy3 before/after miR-21 capture. (**E**) Schematic illustration of the surface charge shifts on Au NP/silicon wafer post ATP capture due to probe conformational changes. (**F**) Surface potential images before/after ATP capture. (**G**) Schematic illustration of the surface charge shifts post miR-21 capture. (**H**) Surface potential images before/after miR-21 capture.

In the aforementioned work, signal-on detection strategies for ATP and miR-21 are successfully established. Then, the above results and hypotheses are validated through a series of characterizations. Firstly, in order to clarify the change in distance between the nucleic acid probe and the substrate before and after the target capturing, surface-enhanced Raman scattering (SERS) analysis which is highly sensitive to the distance between the target and the Au NPs is conducted. As demonstrated in Fig. 3A, the Cy3 molecule is modified at top end of the nucleic acid probe and determined the change in distance between the probe and the substrate through the change in SERS signal intensity of Cy3. The SERS signals of Cy3 molecule obtained from Au NPs-PDMS substrate displays characteristic peaks (*31*) at 1193 and 1586 cm^−1^. When the ATP-probe-Cy3 binds to the target molecule, it can be observed that the SERS intensity of Cy3 increases (Fig. 3B). This indicates that the specific recognition of the target molecule by the probe results in a change in its spatial conformation, which decreases the distance between Cy3 and the substrate. Similarly, when the miR-21-probe-Cy3 binds to the target molecule, the change in probe’s spatial conformation leads to an increase in its distance from the substrate, resulting in a decrease in SERS signal intensity (Fig. 3, C and D).

Then, to study the surface potential change before and after target capturing, we employ Kelvin probe force microscopy (KPFM), which can give information about the electronic state of the surface. The contact potential difference (*V*_CPD_) between the sample and AFM tip is defined as (*32*)

$$V_{\text{CPD}}=V_{\text{sample}}-V_{\text{tip}}$$

where *V*_sample_ and *V*_tip_ are the surface potential of the sample and the AFM tip. Since the surface potential of the probe tip is constant, *V*_sample_ can be compared by measuring surface potential difference. As shown in Fig. 3, E and F, the contact potential difference of the ATP-probe modified surface between AFM tip is 148 mV, after the reaction with 50 nM of ATP, the contact potential difference changed to −227 mV. It is indicated that the surface has a more negative surface potential due to the change of spatial conformation after binding the target molecule, which is consistent with zeta potential results. The miR-21-probe-MB modified surface also shows similar experimental results (Fig. 3, G and H).

### Device performance optimization based on Debye length modulation

**Fig. 4.**
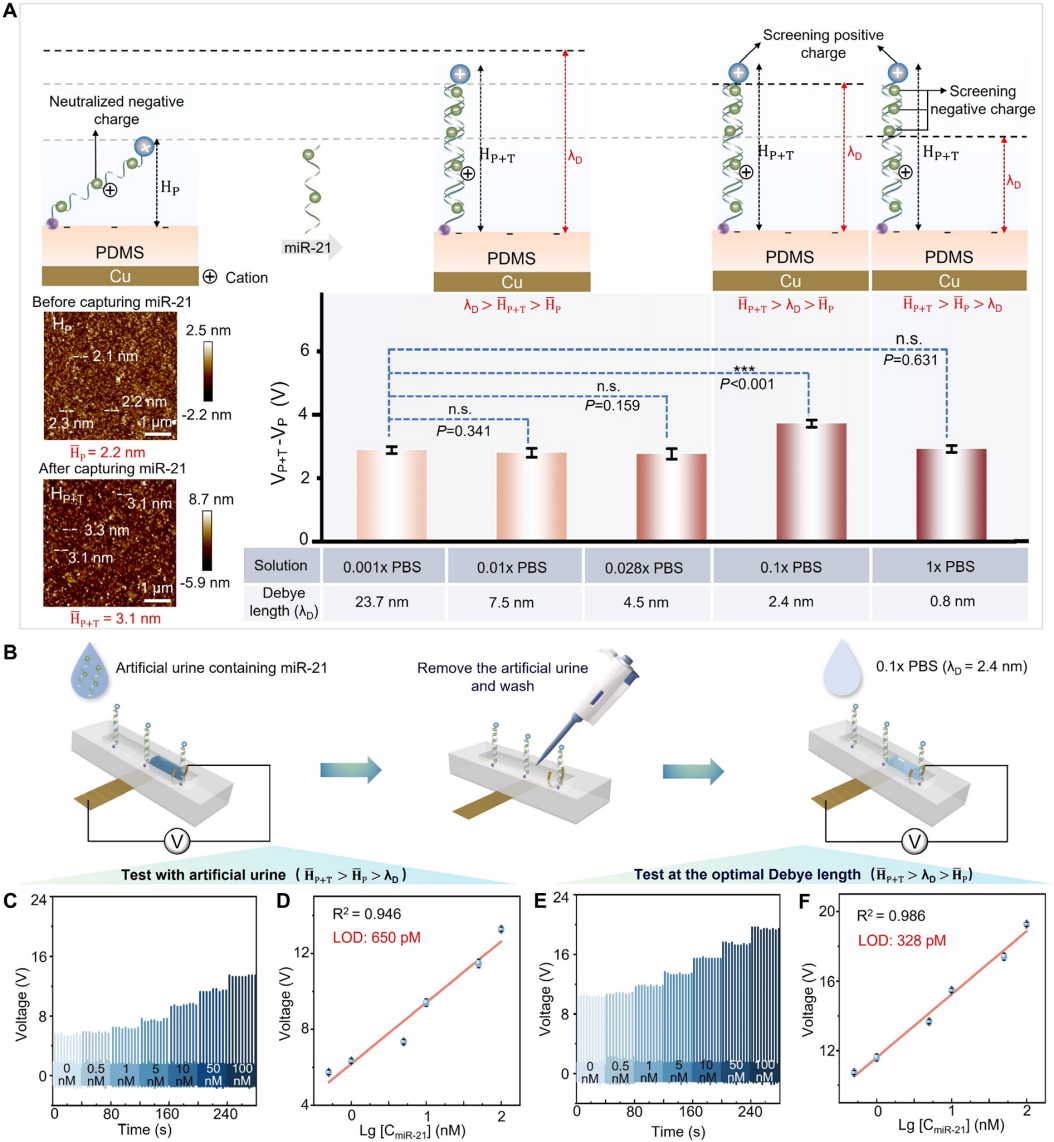
The influence of the Debye length on the detection performance of miR-21. (**A**) The impact on the device’s output electrical signal of adjusting the Debye length through variations in PBS concentration, which results in a relative change in the Debye length compared to the two height values of the probe before and after capturing miR-21. (**B**) Schematic illustrating the process of detection of miR-21 in artificial urine, followed by adjusting the device to operate at the optimal Debye length through the replacement of the test solution. (**C** and **D**) Output voltages of the self-powered sensor, as a function of miR-21 concentration and the corresponding calibration curve. During the test, artificial urine was used as the test solution. (**E** and **F**) After being fully exposed to artificial urine containing miR-21 of different concentrations, the artificial urine was replaced with 0.1 × PBS for testing. Output voltages of the self-powered sensor, as a function of miR-21 concentration and the corresponding calibration curve.

In the aforementioned work, the liquid used for testing before and after the probe captures the target on the device surface is 0.001× PBS. Therefore, in the case of ignoring Debye screening, the influence of nucleic acid probes on the distribution of sensing surface charge before and after capturing the target is effective. However, to transition to more complex solution systems, the Debye screening effect must be considered (Fig. 4A). From the Guoy-Chapman double layer model, we can see that the relationship between λ_D_ and the ionic strength of the solution is as follows (*29*)

$$\lambda_{D}=\sqrt{\frac{\varepsilon_{0}\varepsilon_{r}K_{B}T}{2N_{A}e^{2}I}}$$

In the equation, λ_D_ represents Debye length, which refers to the distance over which the electric field of a charged object can exert an influence, ε_0_ represents the vacuum permittivity, ε_r_ represents the relative permittivity, *K*_B_ represents the Boltzmann constant, *T* represents the thermodynamic temperature, *N*_A_ represents Avogadro’s constant, *e* represents the elementary charge, and *I* represents the ion strength. According to the equation, λ_D_ is mainly determined by the ionic composition of the solution.

In order to study the impact of Debye screening on the sensing performance, PBS solutions with different dilution factors containing the same target molecule concentration are added to the devices, followed by shaking the devices to obtain a series of output electrical signals. The adjustment of the dilution factor of PBS is to regulate the ionic strength of the solution, thereby achieving modulation of the Debye length. Firstly, we investigate the direct impact of salt concentration on the output signal intensity (fig. S14). It is observed that as the salt concentration increases, the output electrical signal strength of the device shows a trend of initially increasing and then decreasing, regardless of whether the target is captured. This is because low salt concentrations are conducive to improving the conductivity of the solution, facilitating charge transfer in the contact electrification process, and enabling the triboelectric material surface to carry more negative charges. However, under high salt concentrations, neutralization and screening effect reduces the negative charge density on the triboelectric material, leading to a decrease in the output electrical signal (*33*).

Next, we compare the difference in output voltage of the devices before and after capturing the same concentration of targets. As it represents the magnitude of signal change that can be achieved with the same concentration of targets. We also investigate how this difference changes with variations in PBS concentration. It is found that there are significant differences between the ATP capture system and the miR-21 capture system.

For miR-21 detection system, at a specific PBS concentration (0.1×), there is a noticeable maximum value in the voltage difference before and after capturing the target. At this point, when the probe captures miR-21, it changes from a fallen-over state (${\bar{H}}_{P}$ = 2.2 nm) to an upright state (${\bar{H}}_{P+T}$ = 3.1 nm), and this causes the height of the MB to be slightly greater than the Debye length (λ_D_ ≈ 2.4 nm). Consequently, the weakening effect of the positive charges carried by the MB on the negative charges on the surface of the triboelectric material is completely screened (Fig. 4A). At this point, since capturing miR-21 increases the total amount of negative charges, the weakening effects of ion-induced charge neutralization and the increased distance between the negatively charged nucleic acid backbone (after capture) and the surface of the triboelectric material on the number of negative charges are both insignificant. In the end, the enhancing factors outweigh the weakening factors, resulting in an increase in the output voltage difference (Fig. 4A). It is noteworthy that AFM characterization has revealed that the height of the probe after capturing the target (H_P+T_) is approximately 3.1 nm, which is significantly lower than the ideal fully upright height expected after capturing miR-21 with nucleic acid probe. The main reason for this is that the density of nucleic acid probes modified on the device surface is not high, and after capturing the target, the nucleic acid probes are not in an ideal upright state but remain at a certain angle with the substrate (*34*).

The importance of comprehending the aforementioned process in detection lies in the capacity to achieve optimum results by substituting urine with a PBS solution of suitable concentration for a second test once the probes have completely captured the target in the urine. Validation through an artificial urine system reveals that when miR-21 is directly detected using artificial urine as the test solution, the detection limit is 650 pM. However, after the probes fully captured miR-21 in the artificial urine, removing the artificial urine, washing, and then performing the detection with 0.1× PBS (Fig. 4B), the detection limit achieved is 328 pM (Fig. 4, C to F). Another advantage of testing with the optimally concentrated PBS solution lies in the further avoidance of the impact on detection caused by fluctuations in salt concentration in real urine samples when confronted with them.

**Fig. 5.**
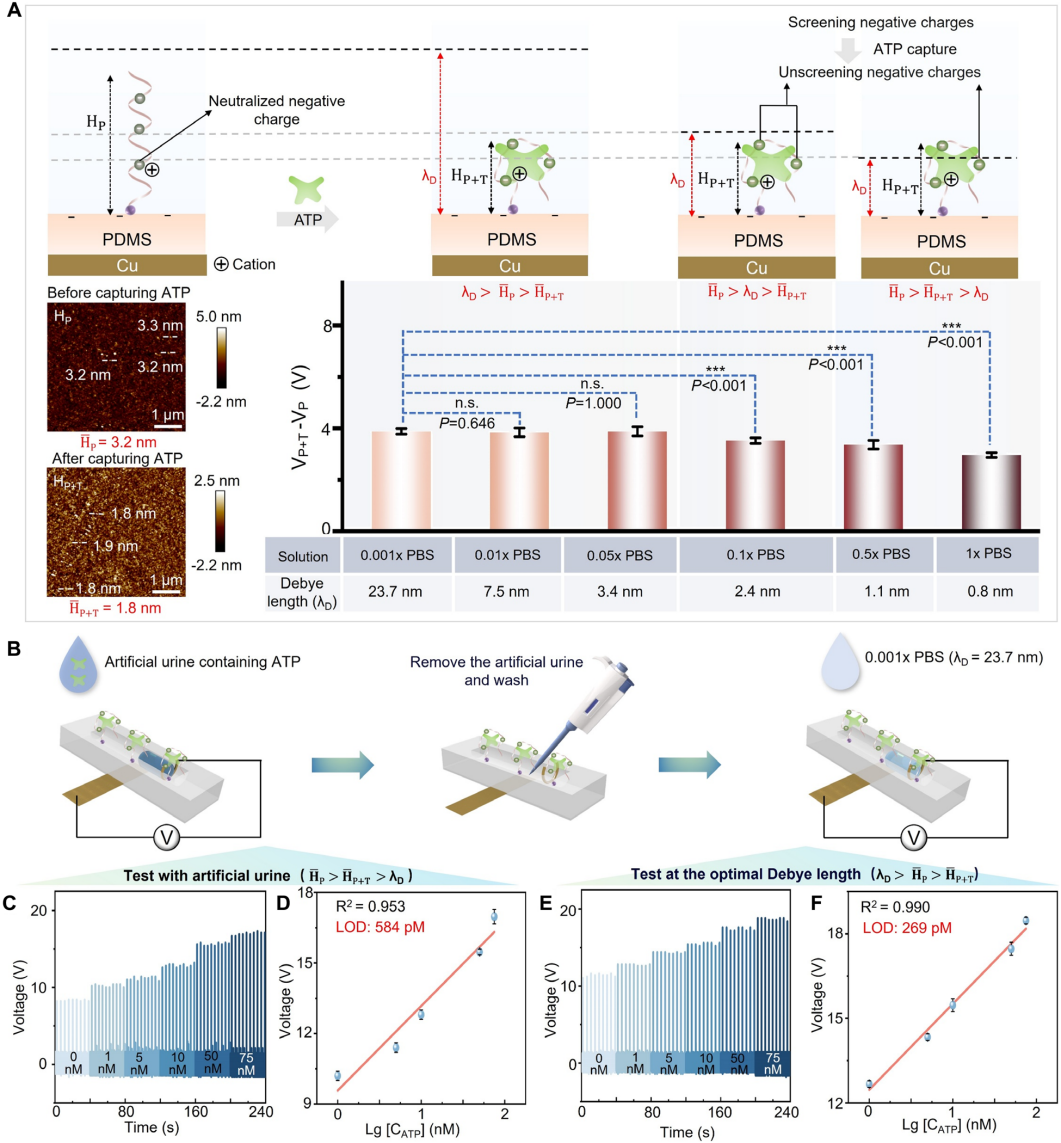
The influence of the Debye length on the detection performance of ATP. (**A**) The impact on the device’s output electrical signal of adjusting the Debye length through variations in PBS concentration, which results in a relative change in the Debye length compared to the two height values of the probe before and after capturing ATP. (**B**) Schematic illustrating the process of detection of ATP in artificial urine, followed by adjusting the device to operate at the optimal Debye length through the replacement of the test solution. (**C** and **D**) Output voltages of the self-powered sensor, as a function of ATP concentration and the corresponding calibration curve. During the test, artificial urine was used as the test solution. (**E** and **F**) After being fully exposed to artificial urine containing ATP of different concentrations, the artificial urine was replaced with 0.001× PBS for testing. Output voltages of the self-powered sensor, as a function of ATP concentration and the corresponding calibration curve.

For ATP detection system, with low concentration PBS, the impact of salt concentration on the difference in output signal is relatively small. Additionally, since the Debye length at this point is greater than the length of the nucleic acid probe, the entire conformational change before and after the nucleic acid probe captures the target can be considered to occur within the Debye length. Therefore, there is no significant difference in the output voltage difference. When using PBS with a concentration of 0.1× or higher, the height of the ATP probe when fully upright (H_P_) is bigger than λ_D_, but after capturing ATP, the height (H_P+T_) is less than λ_D_. In other words, the ATP probe experiences a transition from being partly screened to completely unscreened (Fig. 5A). Theoretically, the output voltage difference before and after capturing should be significant in this situation, but the high concentration of salt ions at this point also strongly weakens the output performance by charge neutralization. Under the competition of these two factors, the output voltage difference still shows a decreasing trend. Similarly, for the ATP detection system, when artificial urine was replaced with 0.001× PBS (Fig. 5B), the detection limit also decreased from 584 pM to 269 pM (Fig. 5, C to F). The results from tests conducted using more complex systems such as PBS and artificial urine further validate the sensing mechanism of the device, as well as its sensitivity and specificity. Furthermore, by conducting secondary detection at the optimal Debye length, the device can achieve even better detection performance. Standardizing the fabrication process can ensure that the device exhibit good repeatability (fig. S15). Meanwhile, after long-term stability testing, the device can maintain good stability over a period of one month (fig. S16). In the future, by designing an integrated microfluidic device, nucleic acid probes can be stored in separate chambers as lyophilized powder, isolated from other solutions, prior to detection, thereby extending their shelf life. During detection, the device can be manually actuated as needed to complete each operational step and the detection. By comparing the detection performance of the developed self-powered device with that of existing portable detection devices or methods, it can be seen that the ATP detection performance of the present device is at a relatively good level (table S1). In particular, its limit of detection is significantly lower than the clinical threshold, demonstrating its potential for clinical urine sample detection.

Due to the complex composition of urine, the anti-interference performance of the device was also investigated. Here, combined with the previous performance comparison, taking the device targeting ATP detection in urine, which holds greater practical application potential, as an example. First, the effect of urea concentration in artificial urine on the test results was discussed. When artificial urine with different urea concentrations was directly used as the triboelectric material during testing, the output signal intensity generated by the devices decreased with increasing urea concentration. This is mainly because the adsorption of urea on the surface of the triboelectric material reduces the number of negative charges on its surface. Subsequently, after removing the artificial urine and cleaning the device, testing was conducted using 0.001× PBS. It can be observed that the effect of urea concentration on the output electrical signal intensity is not significant (fig. S17). This is because the adsorption of urea is not strong, and testing with cleaning and 0.001× PBS can mitigate the interference of urea on the device performance to a certain extent. Subsequently, the effect of pH on the detection performance of the device was investigated through similar procedures. First, the normal pH range of urine is between 5.1 and 6.8 (*35*). Therefore, artificial urine was adjusted within a pH range of 4.5 to 8.0 in this study, and its influence on the output electrical signal intensity of the device was explored (fig. S18). Overall, when the urine is on the acidic side, the output electrical signal intensity decreases significantly due to the neutralization of negative charges on the triboelectric material surface by hydrogen ions. Moreover, since hydrogen ions have a strong adsorption capability, even after cleaning the device and switching to 0.001× PBS for testing, the output performance of the device cannot be fully recovered. Correspondingly, when the urine is on the alkaline side, the output electrical signal intensity increases because it promotes the PDMS surface to carry more negative charges (*14*). However, after cleaning the device and switching to 0.001× PBS for testing, the output performance of the device is largely recovered. These results indicate that urine with an excessively low pH may interfere with the detection performance of the device. After the target is captured and the device is cleaned, switching to 0.001× PBS for testing can mitigate the interference of pH fluctuations on the detection performance to a certain extent. In contrast, since proteins in urine typically carry a positive charge, they readily form strong bonds with the negatively charged triboelectric material surface, thus significantly affecting the device’s detection performance. In such cases, even after cleaning the device and switching to PBS solution, the detection performance is difficult to recover. Therefore, when the device’s detection signal is abnormally low, it is recommended that users conduct a more comprehensive test (fig. S19).

### Testing of clinical urine samples and manually-driven device

**Fig. 6.**
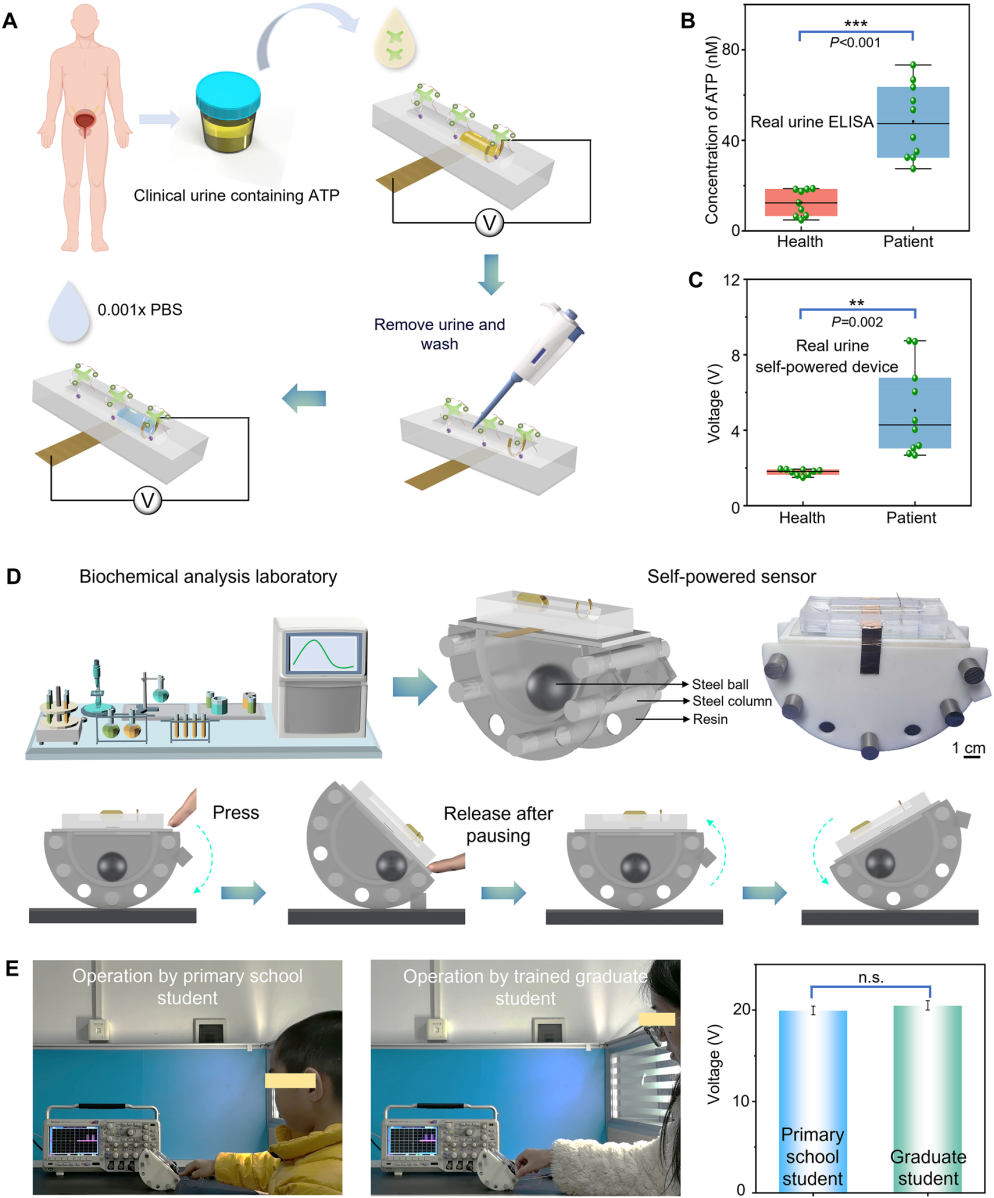
Testing of clinical urine samples and manually-driven device. (**A**) Schematic illustrating the process of detection of ATP in clinical urine samples. (**B**) The ATP concentration in urine samples from both the healthy group and the cystitis patient group was measured through ELISA. (**C**) The output voltage when testing urine samples from both the healthy group and the cystitis patient group using the self-powered sensor. (**D**) A schematic diagram of the further developed manually-driven device. (**E**) Primary school student and graduate student demonstrate the operation of the manually-driven device, along with a differential analysis of the output signals obtained.

Next, the applicability of the present biosensors for detecting biomarkers in real samples is evaluated by specifically detecting the biomarkers in urine samples from patients with cystitis and healthy humans (Fig. 6A). ATP is selected for detection as patients with cystitis are known to typically have elevated levels of ATP in their urine, in contrast to individuals who are healthy. Consequently, the detection of ATP in urine can serve as a means for early diagnosis and monitoring of cystitis (*36*, *37*). First, the concentration of ATP in urine samples from both the healthy group and the cystitis patient group is detected using enzyme-linked immunosorbent assay (ELISA). It is observed that the ATP concentration in urine from cystitis patients is significantly higher than that in healthy individuals (Fig. 6B). Simultaneously, the urine samples are tested using the developed self-powered device. As shown in Fig. 6C, the output voltage of the device that has been in contact with urine from cystitis patients is significantly higher than that of the device that has been in contact with urine from healthy individuals, indicating that this biosensor can effectively respond to ATP in real urine samples and has the potential to achieve early diagnosis of cystitis. Moreover, the intensity of the output electrical signal from the self-powered device also shows a good correlation with the ATP concentration obtained from ELISA tests (fig. S20). Due to the complex components in real urine, the surface of the device’s triboelectric material tends to become hydrophilic (fig. S21). Under such conditions, urine can no longer flow back and forth within the device in the form of droplets, and thus the device’s output performance cannot be guaranteed. Therefore, when testing real urine, the procedure of first capturing in urine, then washing the device, and finally re-measuring in PBS is essential. Finally, to further align with practical applications, we designed a manually driven device that resembles a roly-poly toy and can provide repetitive reciprocating motion. This device enables the liquid sample flow process to be repeatable without requiring an electrical power supply. As shown in Fig. 6D, the device has a limiting device on its right side, which enables the device to stop at a fixed position when pressed. Subsequently, the device is held in this position for 1–2 seconds to allow the solution to come to rest, and then it is released. The solution in the sample cell can then sway along with the device, generating electrical signals. Due to the extremely simple operation of the entire device, both primary school student (7 years old) without specialized training and graduate student can operate it smoothly, and there are no significant differences in the electrical signals obtained (Fig. 6E and movie S2). Meanwhile, this manually driven device also demonstrates good repeatability (fig. S22). Therefore, this device is fully capable of achieving detection through manual driving. This type of self-powered biochemical sensor, owing to its advantages of no need for external power supply, simple structure, low cost, easy to operate as well as high sensitivity and specificity, can perform, to some extent, the tasks of complex biochemical analysis laboratories, thus providing effective diagnostic tools for home health monitoring.

## DISCUSSION

In summary, a self-powered sensor based on nucleic acid probe functionalized double-electrodes TENG for detection of biomarkers in urine is developed. Specifically, the specific detection is achieved by regulating the charge distribution on the triboelectric material surface through the spatial conformational changes caused by nucleic acid probe before and after capturing the target. The sensing mechanisms of two biochemical biomarkers are thoroughly discussed. The device’s capacity to monitor biomarkers in artificial urine such as ATP or miR-21 is effectively demonstrated by test results. Subsequently, the self-powered sensor is successfully utilized to detect abnormally elevated levels of ATP in the urine of patients with cystitis. Therefore, the efficiency and sensitivity of this detection method offer the possibility of developing non-invasive diagnostic tools, which are expected to realize high-sensitivity, and high-specificity detection of biomarkers in urine. Ultimately, our present work provides a potential low-cost, portable, easy to operate, battery-free biochemical marker detection device that can be applied even in environments with limited resources. If more specific probes can be introduced to expand the range of detectable targets, this type of self-powered device is expected to play a significant role in home health monitoring.

## MATERIALS AND METHODS

### Reagents and materials

Polydimethylsiloxane (PDMS) prepolymer and curing agent were bought from Dow Corning, USA. Glass slide with a length of 76.2 mm, width of 25.4 mm and thickness of 1.1 mm was bought from Suzhou shenying optical Company, China. Conductive Cu wire with a diameter of 0.4 mm were purchased from Qinghe shenghang metal material Company, China. Conductive Cu tapes with a thickness of 60 μm were purchased from Shenzhen Mileqi Company, China. Chloroauric acid trihydrate and trisodium citrate dihydrate were purchased from Sinopharm Chemical Reagent Company, China. Tris (2-carboxyethyl) phosphine hydrochloride (TCEP), PBS buffer solution and aptamers were purchased from Shanghai Sangon Biotech Company, China. Deionized (DI) water was used throughout. The sequence of ATP aptamer is: GGGGGAGTATTGCGGAGGA (*38*). The sequence of miR-21 is: UAGCUUAUCAGACUGAUGUUGA. The sequence of nucleic acid probe for miR-21 is: TCAACATCAGTCTGATAAGCTAAAAA. The sequence of mismatch RNA is: UUGUACUACACAAAAGUACUG.

### Fabrication of the fluidic device

PDMS mortar with a 10:1 mixing ratio of base to curing agent was degassed for 30 min in a vacuum jar before spraying. The width of 1 cm copper foil was attached as the bottom electrode in the middle of the glass and placed in a culture dish. Then, the copper wire with a diameter of 0.4 mm is fixed to one end of the acrylic rod that measures 1 cm in diameter and 5 cm in length. The acrylic rod with the copper wire was placed in the middle of the glass. Afterwards, PDMS mortar was poured into a culture dish and subjected to a 3-hour curing process at 60°C. Subsequently, the acrylic rod was removed from the PDMS. Half of the copper wire was embedded in PDMS, thus remaining as the top electrode. During the experiment, the device was fixed on a rotary mixer. The rotation of the device causes the fluid to move back and forth. The flowing liquid can generate corresponding triboelectric signals between the top and bottom electrode.

### Modification of Au NPs

In order to perform gold particle modification, Au NPs synthesis was first conducted. Briefly, 0.025 g of HAuCl_4_·3H_2_O was dispersed in 200 ml of DI water and were heated to boiling point and continuously stirred for 10 min. Then, 1.5 ml 1% sodium citrate solution was added and stirred for 20 min. A change in the solution color to purple indicates the successful preparation of Au NPs. The Au NPs-PDMS was prepared according to the literature. The prepared Au NPs solution was poured into the PDMS fluid channel and incubated at room temperature for 6 h. The prepared Au NPs-PDMS washed with DI water for several times and stored for further use.

### Functionalization of nucleic acid probes

Firstly, the nucleic acid probe (100 μM) and TCEP (20 mM) was incubated 1 hour at room temperature, and further diluted by 1× PBS buffer. Then, the mentioned above solution was added to the PDMS fluid modified with Au NPs, incubated at room temperature for 10 h. The prepared device washed with DI water for several times and stored for further use.

### Characterization

The output currents and voltages were performed by a low-noise current preamplifier (SR570, Stanford Research Systems, Inc., Sunnyvale, CA, USA) and a digital phosphor oscilloscope (MSO 2024B, Tektronix, Inc., Beaverton, OR, USA). The charge density from the output signals was measured using an electrometer (Keithley 6514, Cleveland, OH, USA). The zeta potential (ζ) of the PDMS surfaces at each stage were determined using a SurPASS Electrokinetic Analyzer (Anton Paar GmbH, Austria). During the experiments, a background electrolyte of 1 mM KCl solution was utilized. The morphologies of as-prepared Au NPs-PDMS were analyzed with a field-emission scanning electron microscope (FE-SEM) (S-4800, Hitachi, Tokyo, Japan). The characteristic peaks of Au NPs-PDMS were monitored on an UV-vis spectrophotometer (UV-2600, Shimadzu). The SERS spectra were recorded on a confocal microprobe Raman system (Renishaw, inVia). The excitation wavelength was 633 nm laser is usually from the HeNe laser. During the SERS testing process, the aptamer-Au-PDMS was initially placed in a matrix for testing. Then, the position remained unchanged, the target molecule was added to the matrix and stood for 10 min. Excess liquid was subsequently removed, and SERS testing was conducted at the same location, which ensured the accuracy of monitoring aptamer conformational change during the SERS testing process. The contact potential differences were recorded by a MultiMode 8-HR AFM (Bruker Corporation, Germany).

### Real sample test

Urine samples were collected from 9 healthy volunteers and 10 patients with cystitis. Before detection, the real urine is centrifuged at 10,000 rpm for 3 minutes to remove visible interfering substances. Firstly, the ATP content in urine was determined using ELISA according to the instructions of the ATP detection kit (Beyotime, China). Luminance was measured by microplate reader (Life Technologies Holdings Pte. Ltd. Singapore). On the other hand, urine is dripped into the self-powered device, and the device is shaken back and forth for 10 minutes to ensure the complete binding between the nucleic acid probes and targets. After thoroughly cleaning and drying the device with nitrogen gas, an equal amount of 0.001× PBS was added to the device, which was then shaken again while the output voltage was recorded. The study was approved by the Ethics Committee of Union Hospital, Tongji Medical College, Huazhong University of Science and Technology (approval number: 2020–0453). All the participants in this study signed informed consent forms before participating.

## Supplementary Materials

### The PDF file includes:

Figs. S1 to S22 Table S1 Legends for movies S1 and S2 References
