# Supplementary material for: Nucleic acid probe-functionalized self-powered sensor for detection of biomarkers in clinical urine samples
Source: Sci Adv. 2026 Aug 5;12(32):eaeg5086. doi: 10.1126/sciadv.aeg5086 (PMC13440410; doi:10.1126/sciadv.aeg5086)
Supplement: Supplementary file 1 [file sciadv.aeg5086_sm.pdf]

Science Advances
AAAS

Supplementary Materials for

Nucleic acid probe-functionalized self-powered sensor for detection of biomarkers in clinical urine samples

Qitao Zhou *et al.*

Corresponding author: Qitao Zhou, zhouqitao@cug.edu.cn; Caofeng Pan, 11513@buaa.edu.cn; Fan Xia, xiafan@cug.edu.cn

Sci. Adv. **12**, eaeg5086 (2026)
DOI: 10.1126/sciadv.aeg5086

The PDF file includes:

Figs. S1 to S22
Table S1
Legends for movies S1 and S2
References

Other Supplementary Material for this manuscript includes the following:

Movies S1 and S2

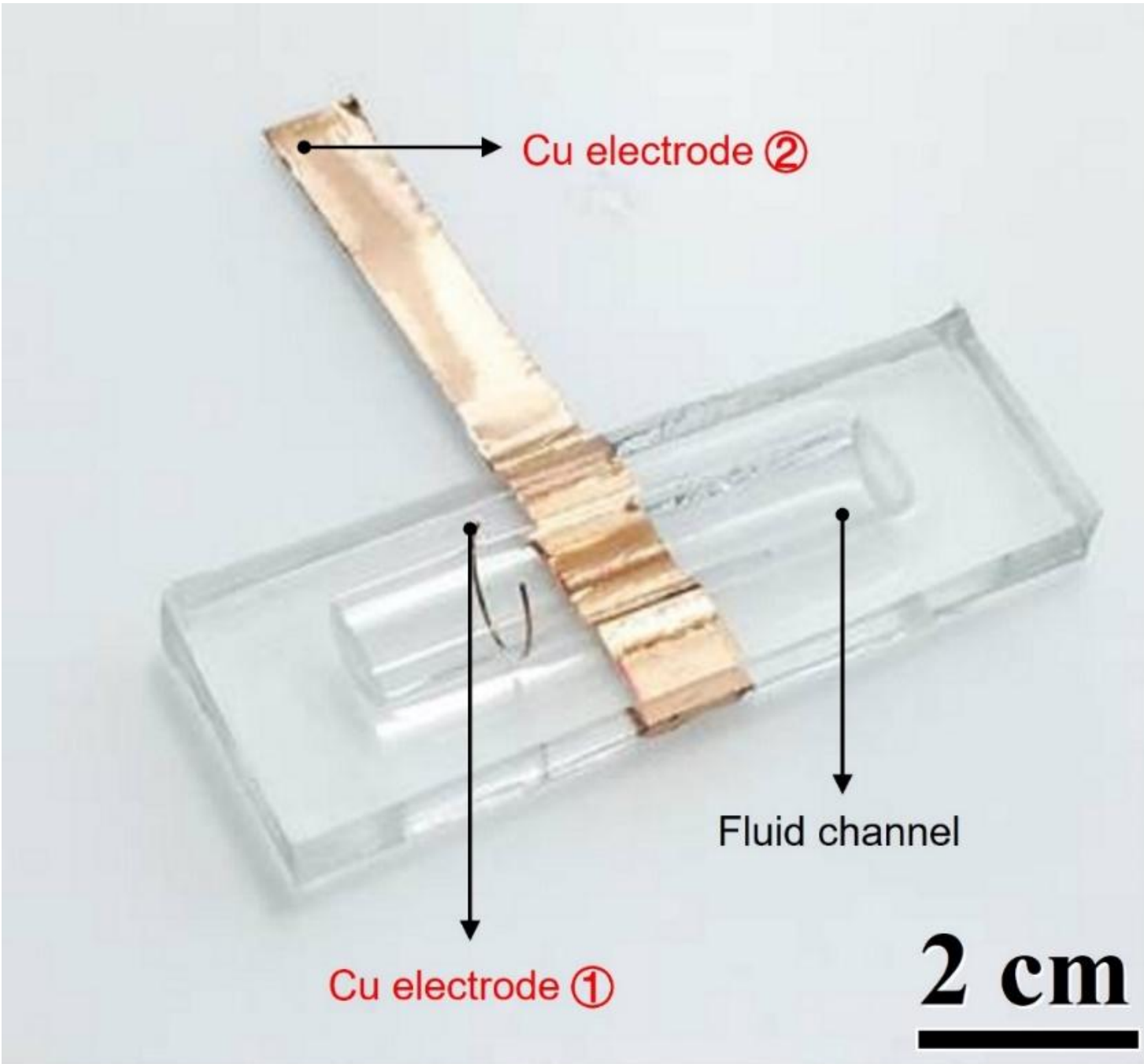

Figure S1. Photograph of the self-powered sensor constructed in this paper.

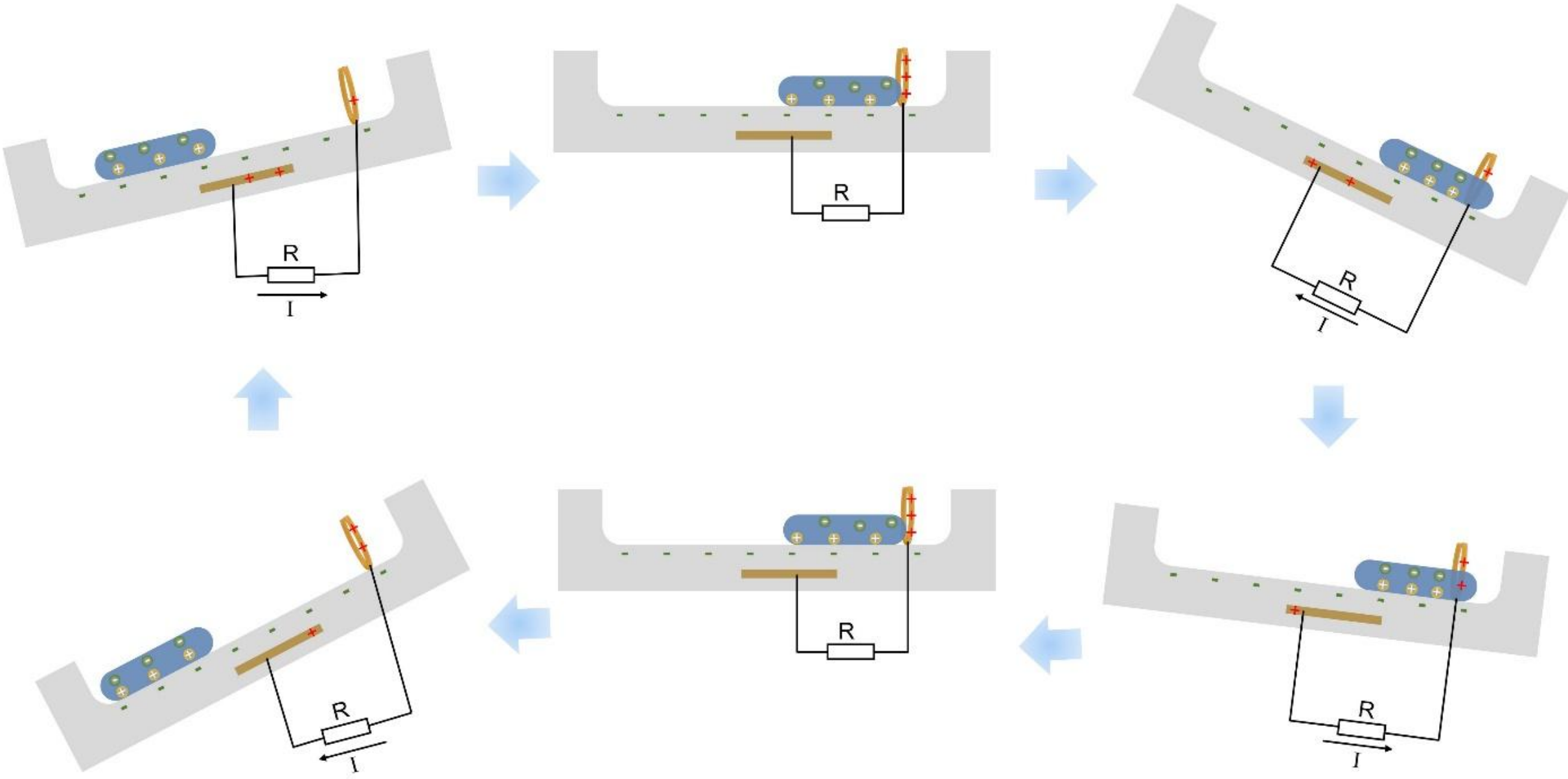

Figure S2. Schematic illustration of the mechanism by which the self-powered sensor generates electrical signals as the solution flows through it.

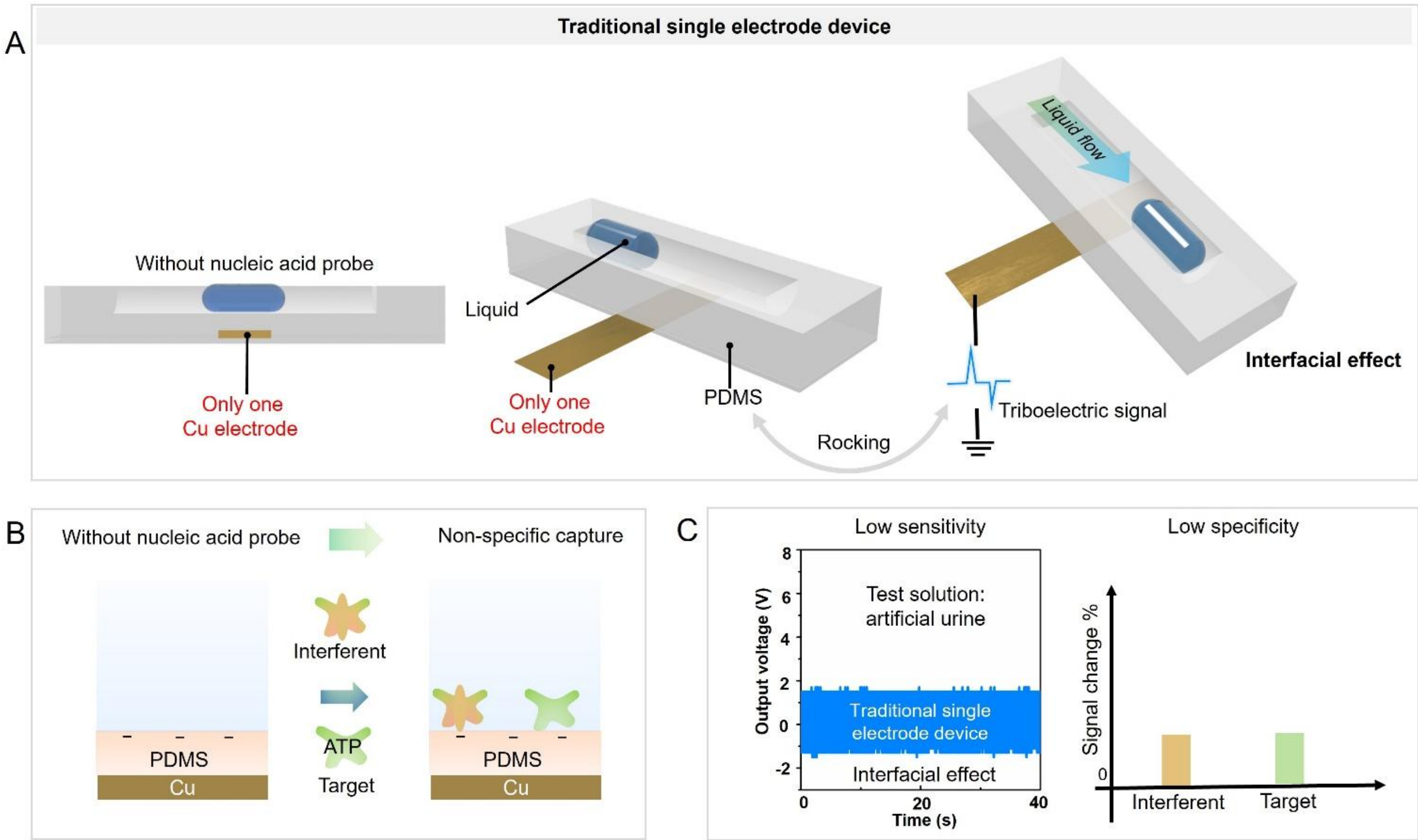

Figure S3. Traditional self-powered sensor based on single-electrode TENG. (A) Structural of the traditional single electrode device. (B) The target capture mechanism of the traditional single electrode device without nucleic acid probe. (C) The detection sensitivity and specificity of the traditional single electrode device.

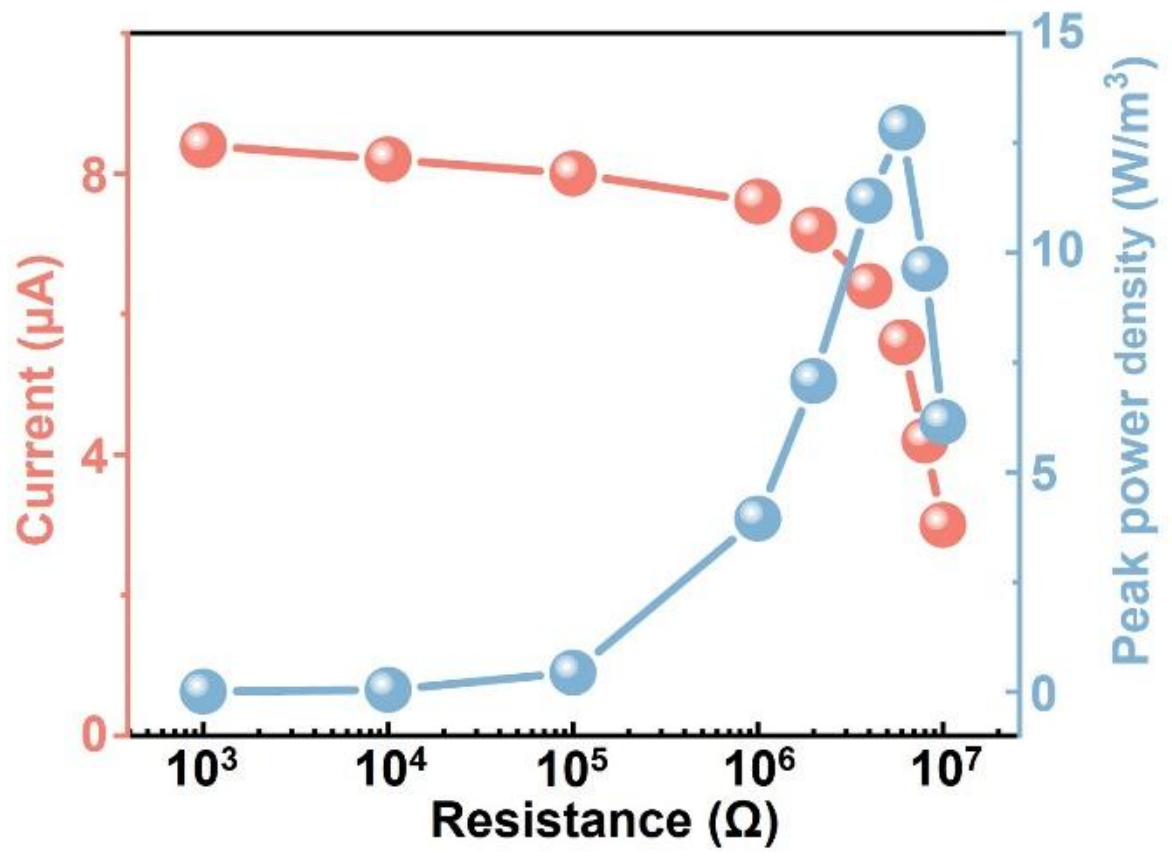

Figure S4. Evolution of current peak and power peak with the increase of external resistance for the device with artificial urine.

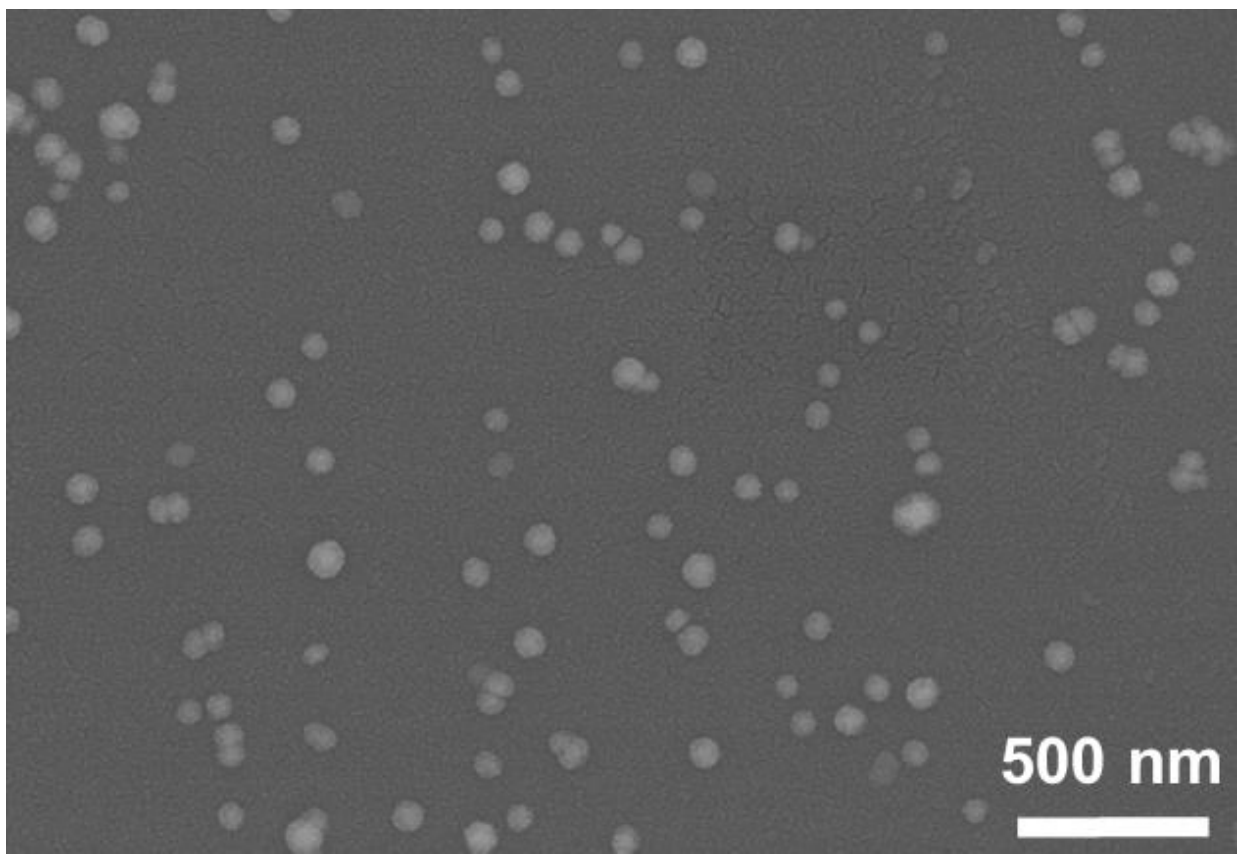

Figure S5. SEM image of the Au NPs-PDMS film.

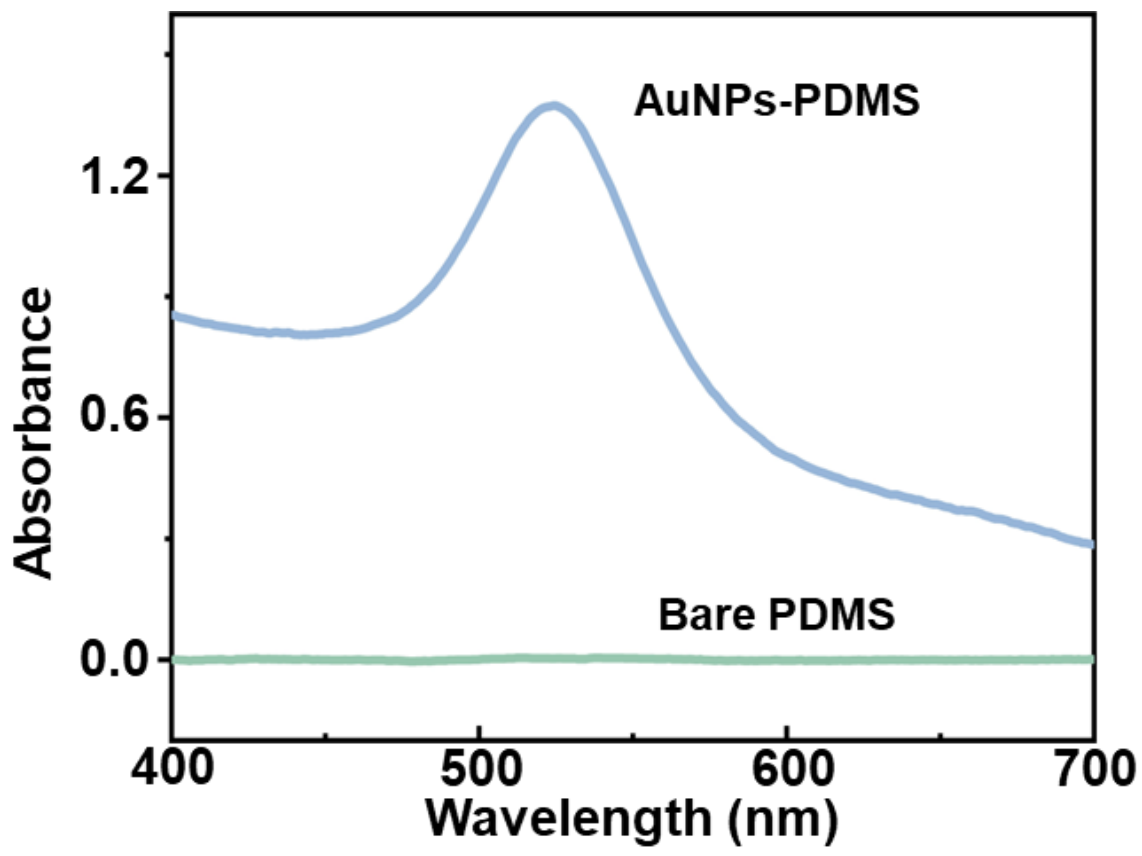

Figure S6. UV-vis absorption spectra of PDMS film and Au NPs-PDMS film.

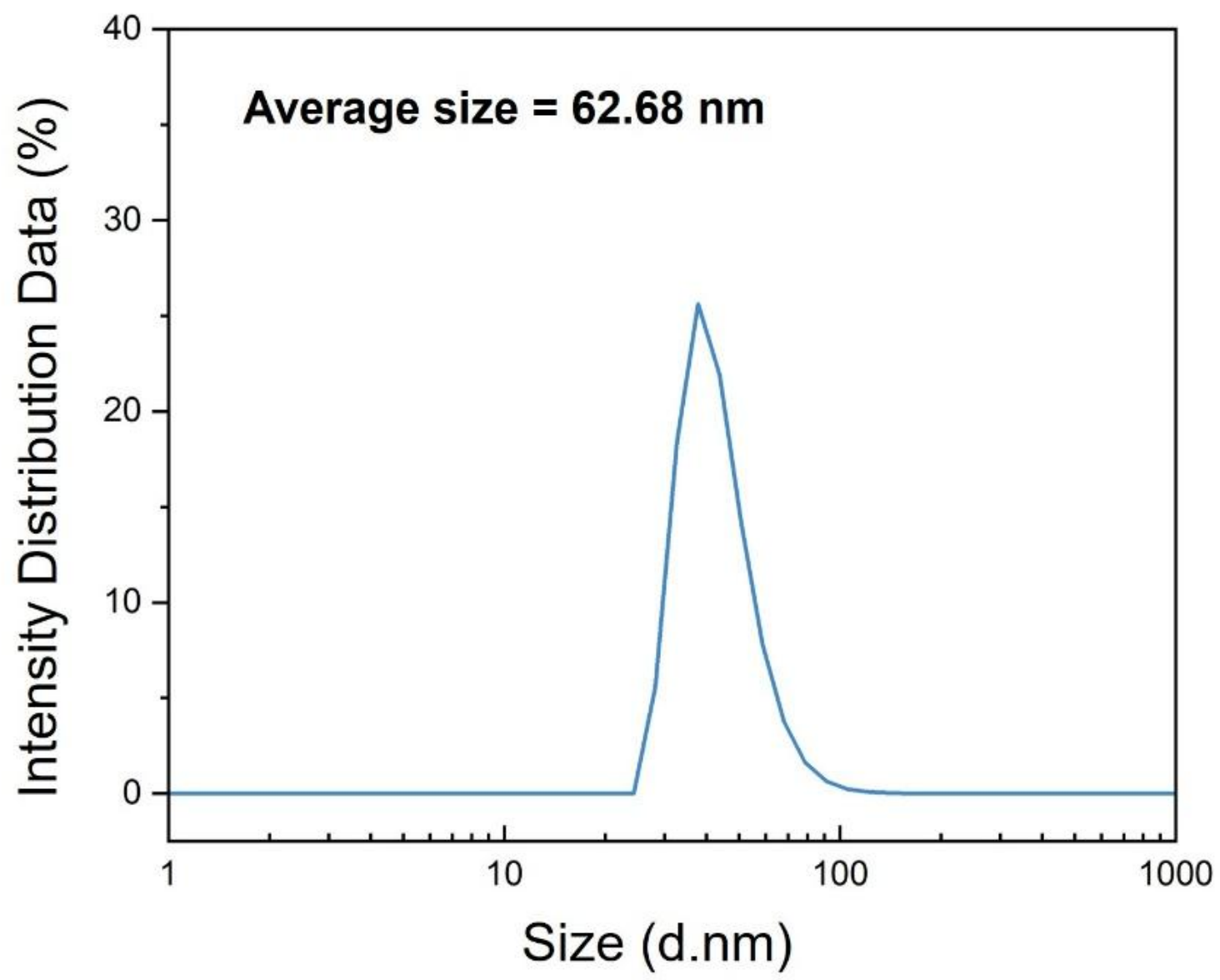

Figure S7. The particle size distribution of the prepared Au NPs obtained through dynamic light scattering measurement.

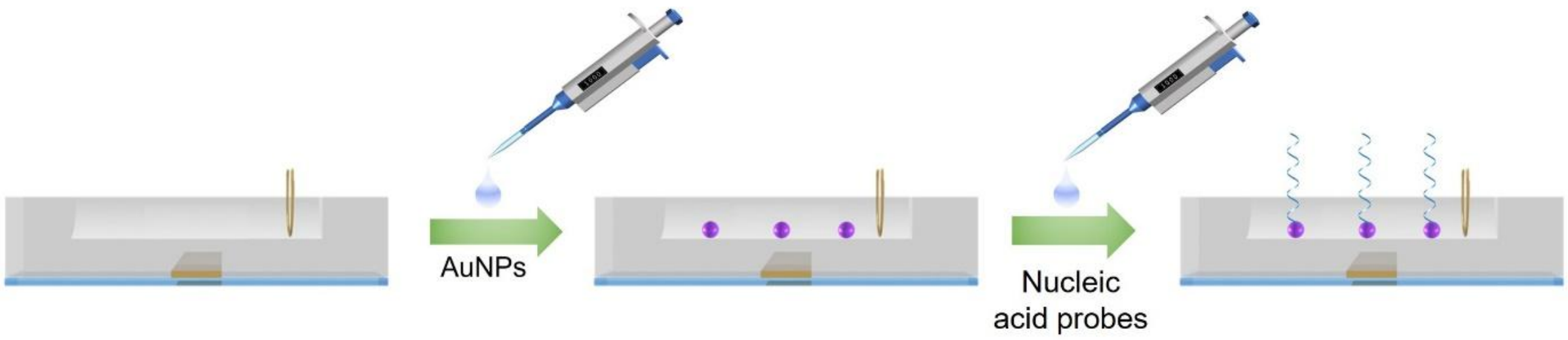

Figure S8. Schematic diagram of the steps for surface modification of nucleic acid probes on the triboelectric material of the device.

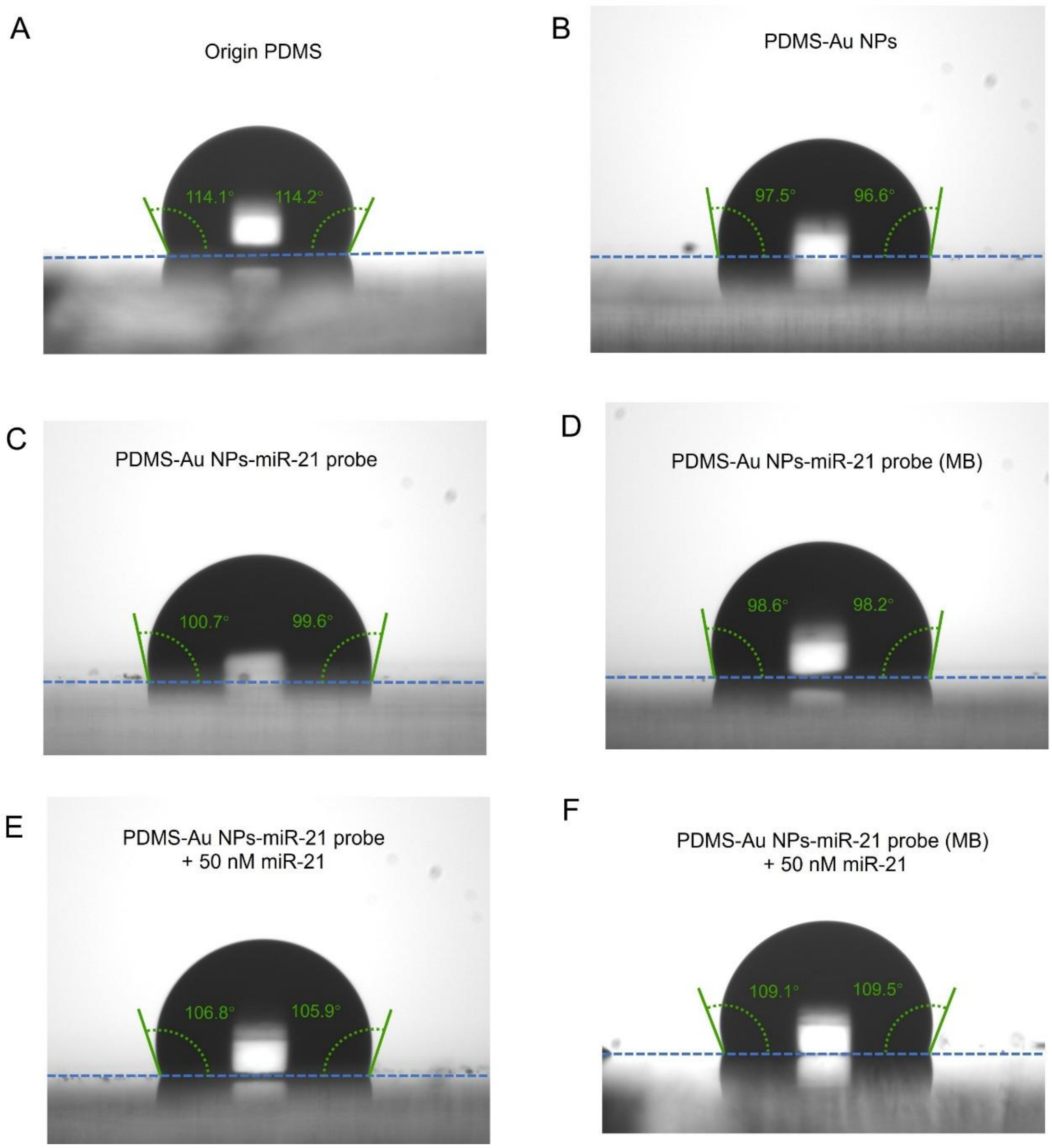

Figure S9. Contact angle measurements of the triboelectric material surface at each stage.

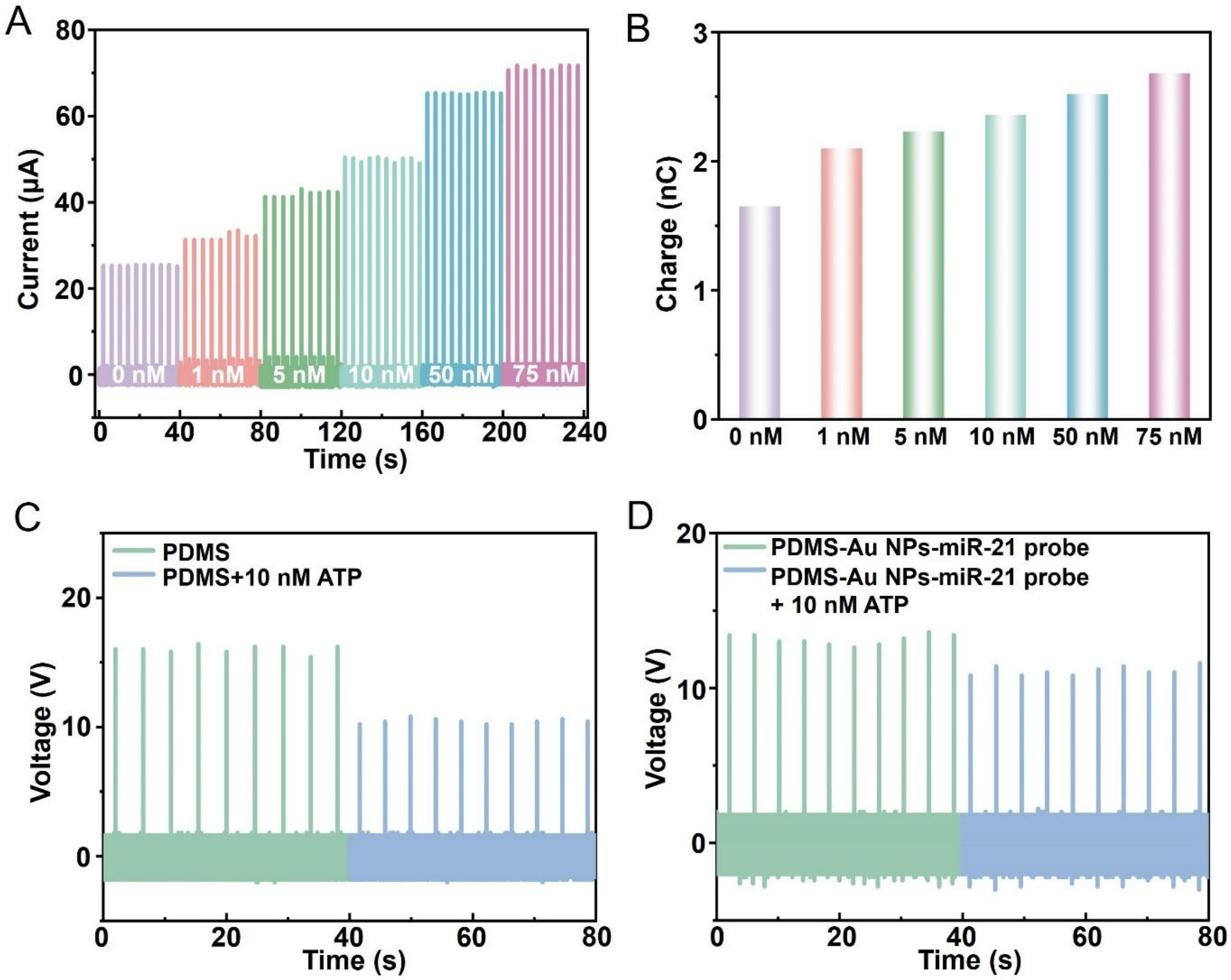

Figure S10. Performance characterization of ATP detection devices. The current (A) and transfer charge (B) of the sensor modified with nucleic acid probe treated with different concentrations of ATP solutions. All the electrical output signals were measured after the device was treated with ATP solutions of different concentrations, then the ATP solution was discarded and the device was cleaned, followed by the addition of an equal volume of 0.001× PBS, and the device was shaken at the same frequency. (C) The device with origin PDMS and its interaction with 0.001× PBS produce a voltage output, as well as the voltage output when the device is fully exposed to 10 nM ATP and then interacts with 0.001× PBS. (D) The device modified with nucleic acid probes targeting miR-21(without MB), and its voltage output when interacting with 0.001× PBS, as well as the voltage output after the device is fully exposed to 10 nM ATP and then interacts with 0.001× PBS.

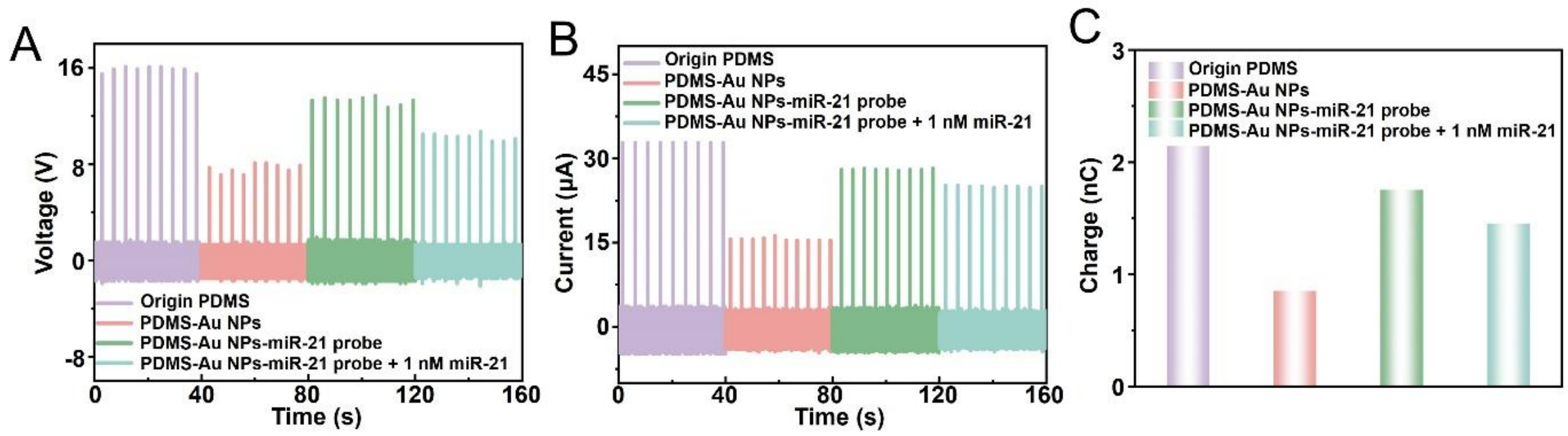

Figure S11. Performance characterization of self-powered sensors modified with miR-21-specific nucleic acid probes that without MB. The output voltage (A), current (B) and transfer charge (C) generated from different preparation steps of the sensors. All electrical output signals are measured after the device has undergone different treatments, followed by cleaning the device, then adding an equal amount of 0.001× PBS to the device, and shaking the device at the same frequency.

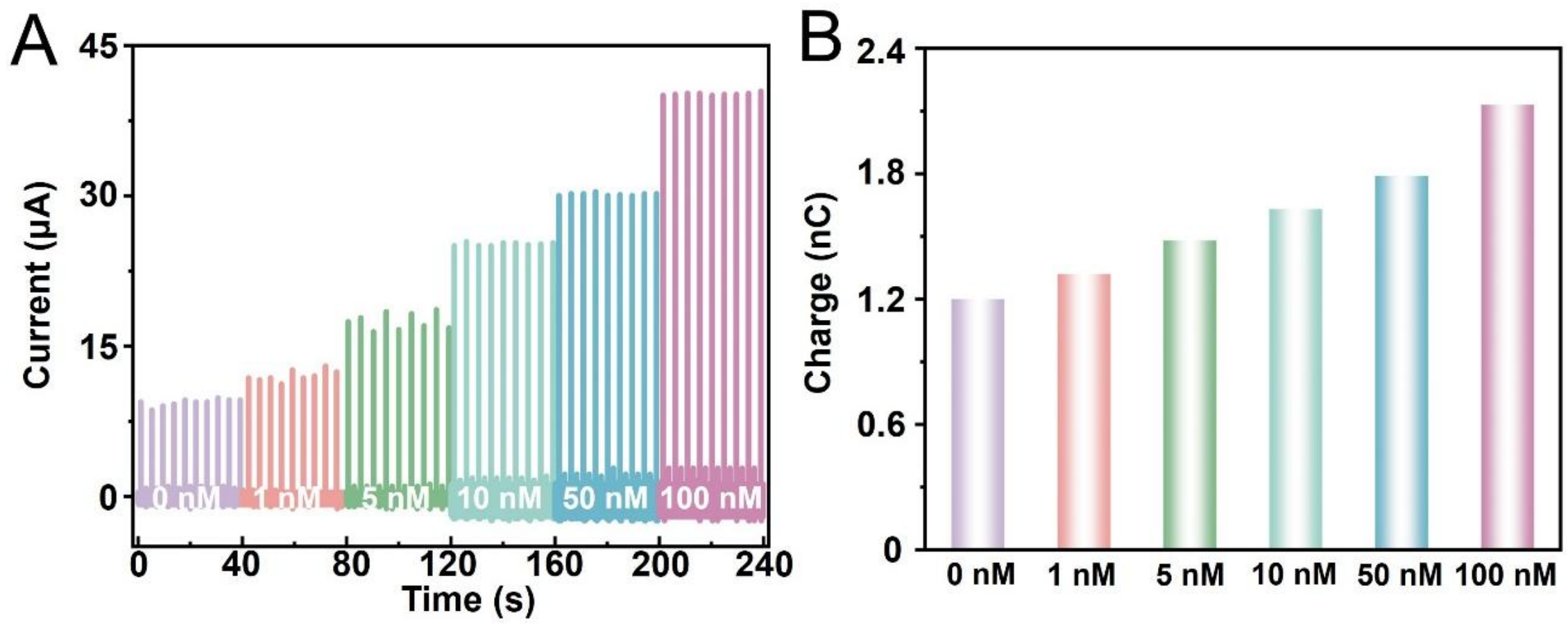

Figure S12. Performance characterization of self-powered sensors modified with miR-21-specific nucleic acid probes that contain MB. The current (A) and transfer charge (B) of the sensor modified with nucleic acid probe treated with different concentrations of miR-21 solutions. All the electrical output signals were measured after the device was treated with miR-21 solutions of different concentrations, then the miR-21 solution was discarded and the device was cleaned, followed by the addition of an equal volume of 0.001× PBS, and the device was shaken at the same frequency.

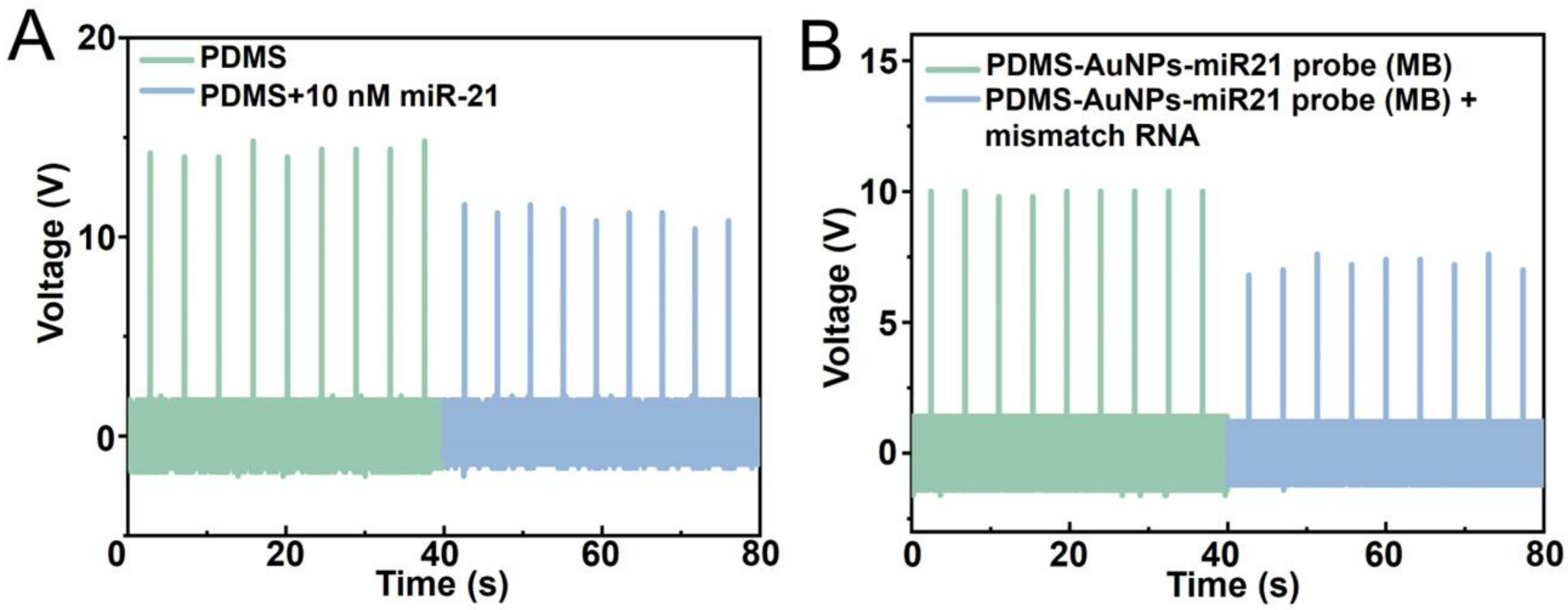

Figure S13. Specificity of the self-powered devices for miR-21 detection. (A) The device with origin PDMS and its interaction with 0.001× PBS produce a voltage output, as well as the voltage output when the device is fully exposed to 10 nM miR-21 and then interacts with 0.001× PBS. The output voltages of the unmodified nucleic acid probe sensor, as a function of water and 10 nM miR-21. (B) The device modified with nucleic acid probes targeting miR-21 (with MB) and its interaction with 0.001× PBS produce a voltage output, as well as the voltage output when the device is fully exposed to 10 nM mismatched RNA and then interacts with 0.001× PBS.

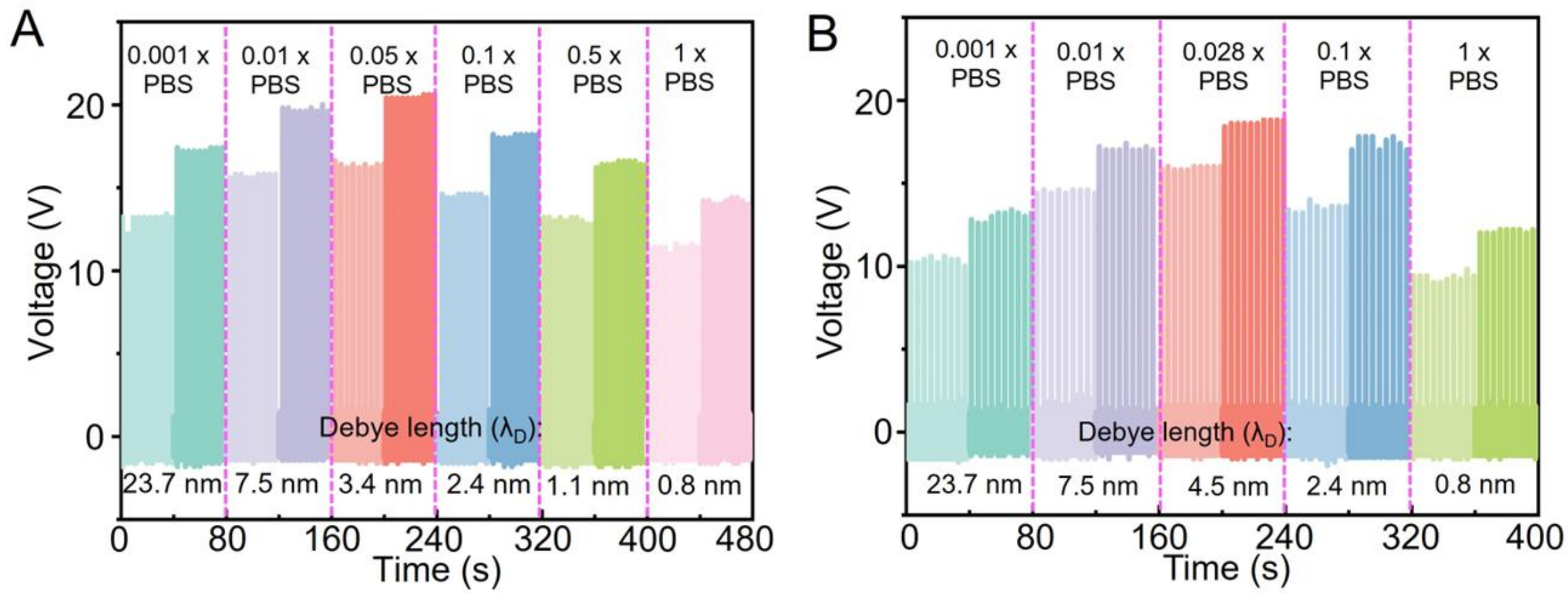

Figure S14. The influence of the Debye length on the output voltage of the device before and after capturing the target. (A) Using PBS solutions with different dilution levels as test solutions, the output voltage of corresponding PBS solutions containing 10 nM ATP were tested using self-powered sensors modified with ATP aptamer. (B) Using PBS solutions with different dilution levels as test solutions, the output voltage of corresponding PBS solutions containing 10 nM miR-21 were tested using self-powered sensors modified with nucleic acid probe (with MB). All the output voltage signals mentioned above were directly measured using PBS buffer solutions with equal volumes but different dilution factors, containing a fixed concentration of target molecules, or without target molecules, and employing a device with a fixed frequency of oscillation.

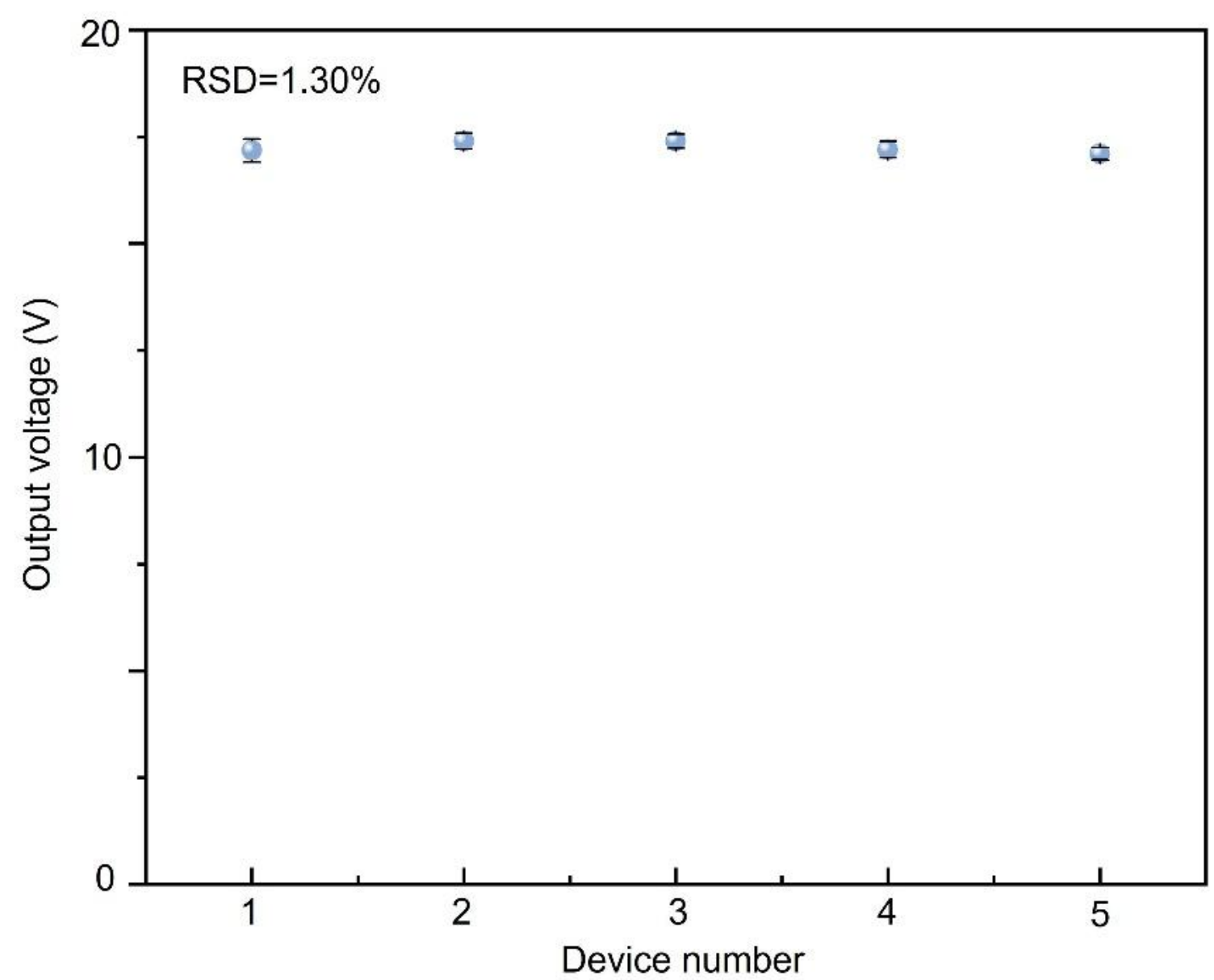

Figure S15. Repeatability of the device for artificial urine testing. The test was performed using artificial urine containing 50 nM ATP as the test sample. The test signal was obtained by replacing the artificial urine with 0.001× PBS solution as the testing solution after the device had fully captured the targets.

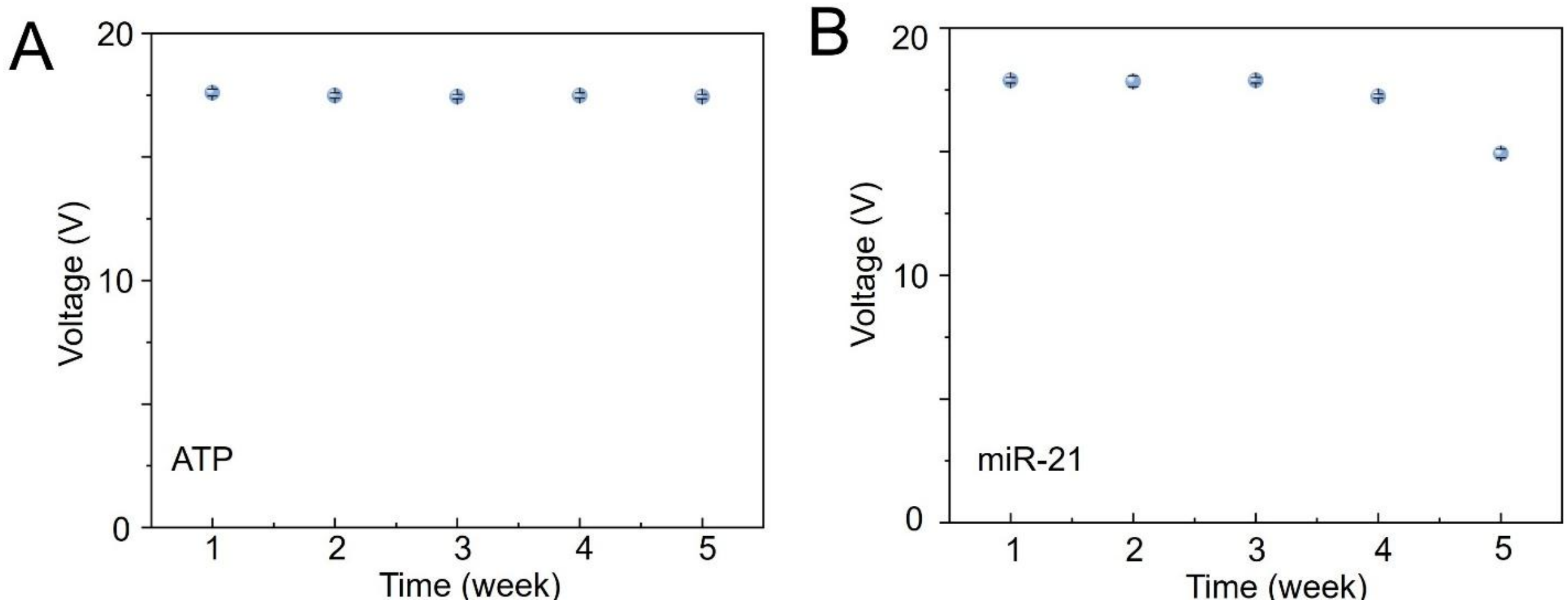

Figure S16. Long-term stability of the devices. (A) The test was performed using artificial urine containing 50 nM ATP as the test sample. The test signal was obtained by replacing the artificial urine with 0.001× PBS solution as the testing solution after the device had fully captured the targets. (B) The test was performed using artificial urine containing 50 nM miR-21 as the test sample. The test signal was obtained by replacing the artificial urine with 0.1× PBS solution as the testing solution after the device had fully captured the targets.

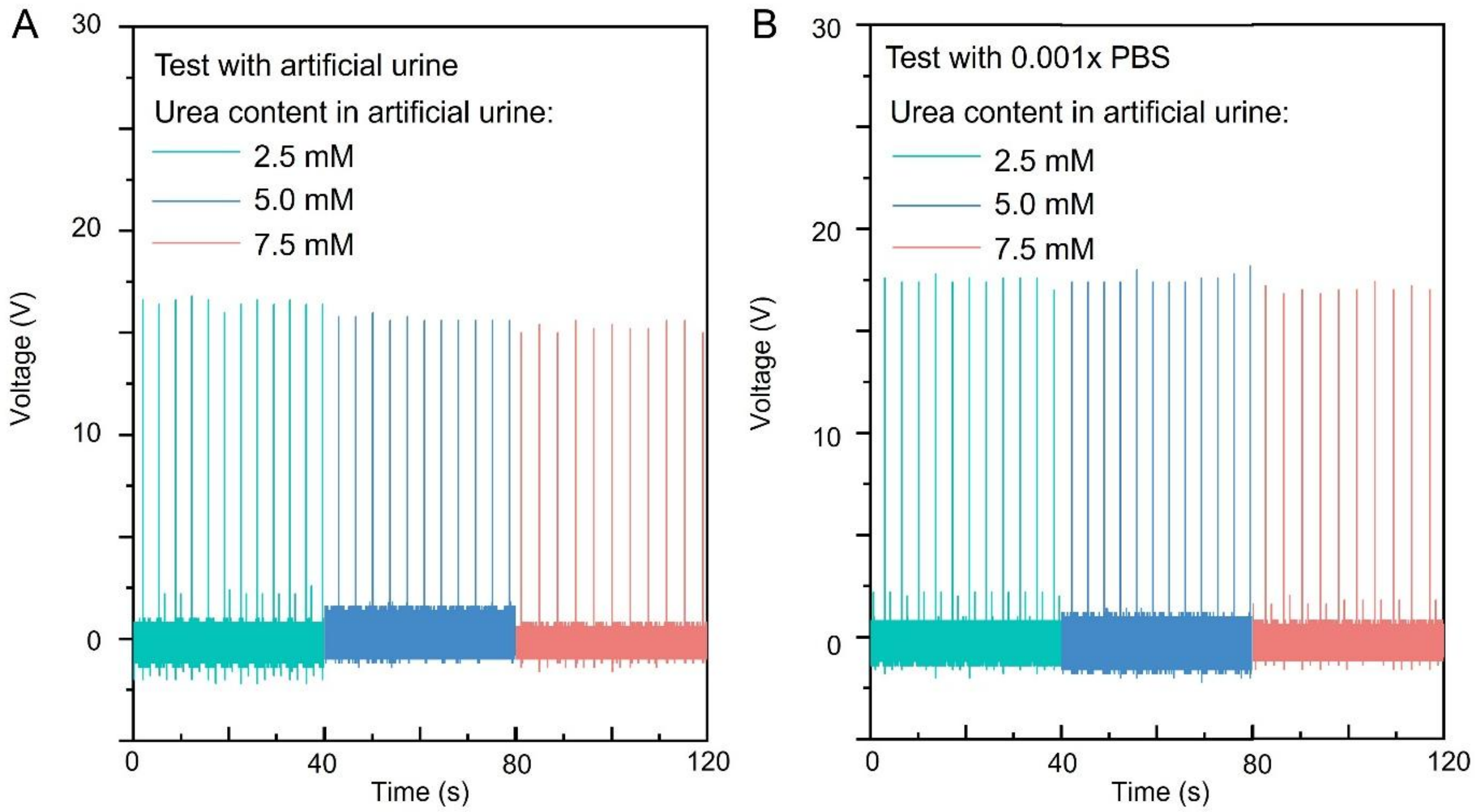

Figure S17. Effect of urea concentration in artificial urine on the output performance of the device. (A) Direct measurement using artificial urine. (B) Output performance measured with 0.001× PBS solution after washing the devices that were soaked in artificial urine containing different urea concentrations. All artificial urine samples used for testing had identical compositions except for the urea concentration, with an ATP concentration of 50 nM.

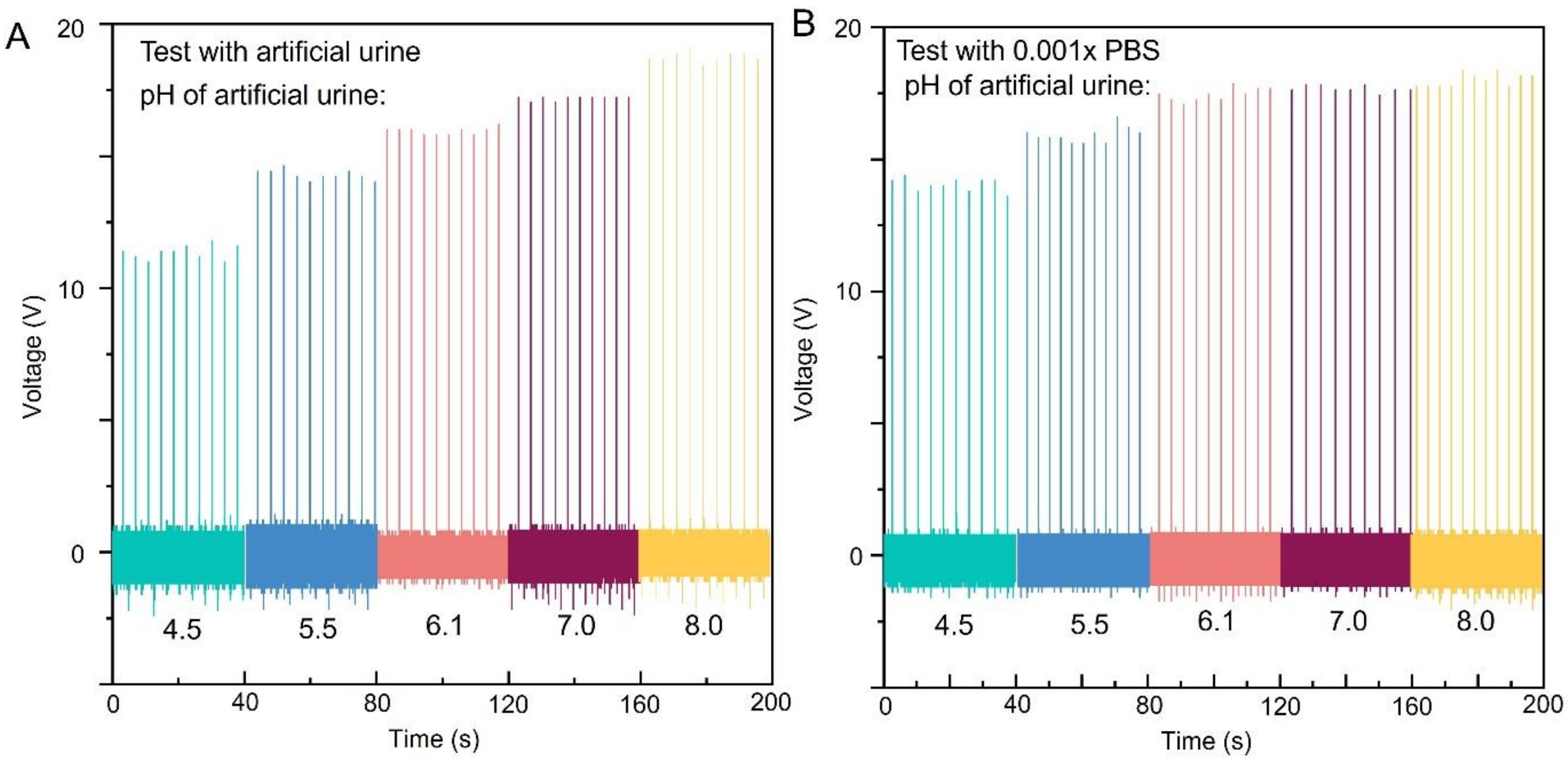

Figure S18. Effect of artificial urine pH on the output performance of the device. (A) Direct measurement using artificial urine with different pH values. (B) Output performance measured with 0.001× PBS solution after washing the devices that were soaked in artificial urine with different pH values. All artificial urine samples used for testing had identical compositions except for pH, which was adjusted by dropwise addition of HCl and NaOH solutions, with an ATP concentration of 50 nM.

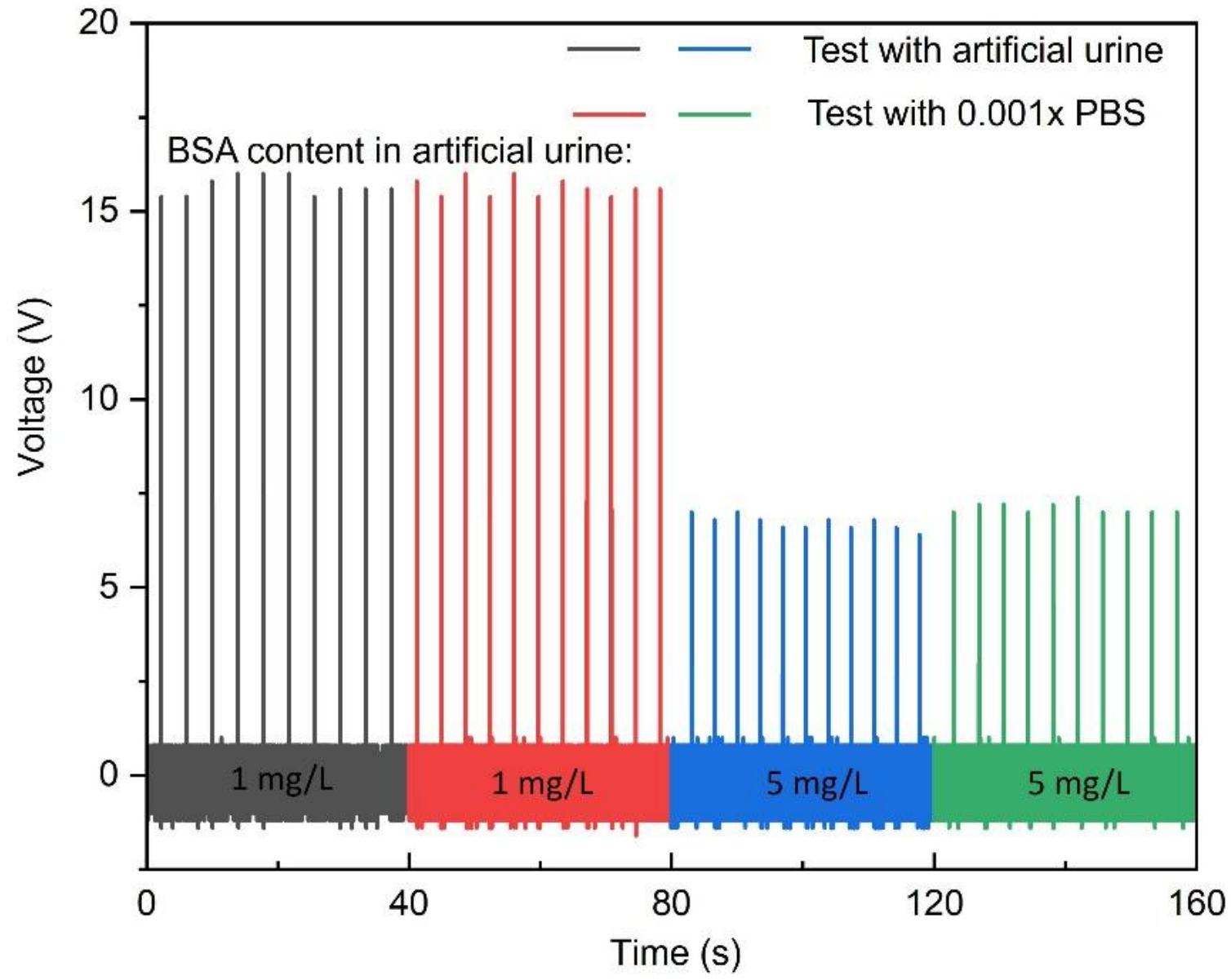

Figure S19. Effect of bovine serum albumin (BSA) concentration in artificial urine on the detection signal intensity of the device.

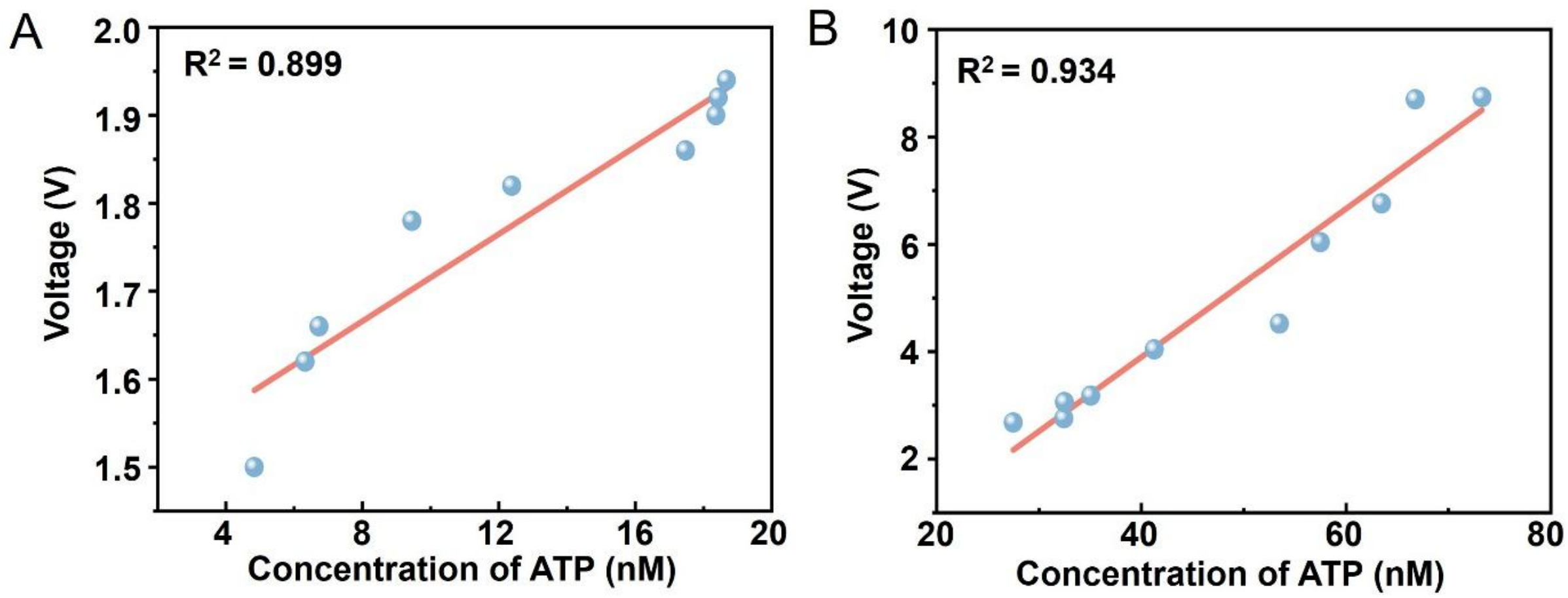

Figure S20. The correlation between the test results of the self-powered device and those of ELISA. (A) The correlation between the concentrations of ATP (measured through ELISA) and output voltages from the self-powered sensor for the healthy group. (B) The correlation between the concentrations of ATP (measured through ELISA) and output voltages from the self-powered sensor for the cystitis patient group.

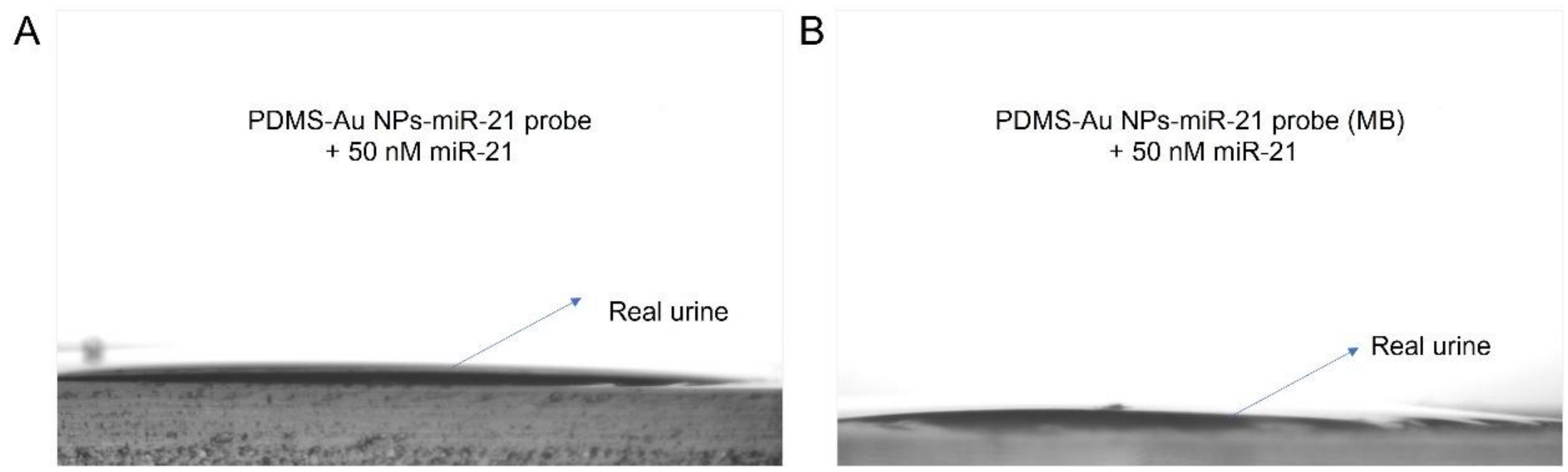

Figure S21. Contact angle measurements of real urine dropped onto the surface of the target-captured triboelectric material surfaces.

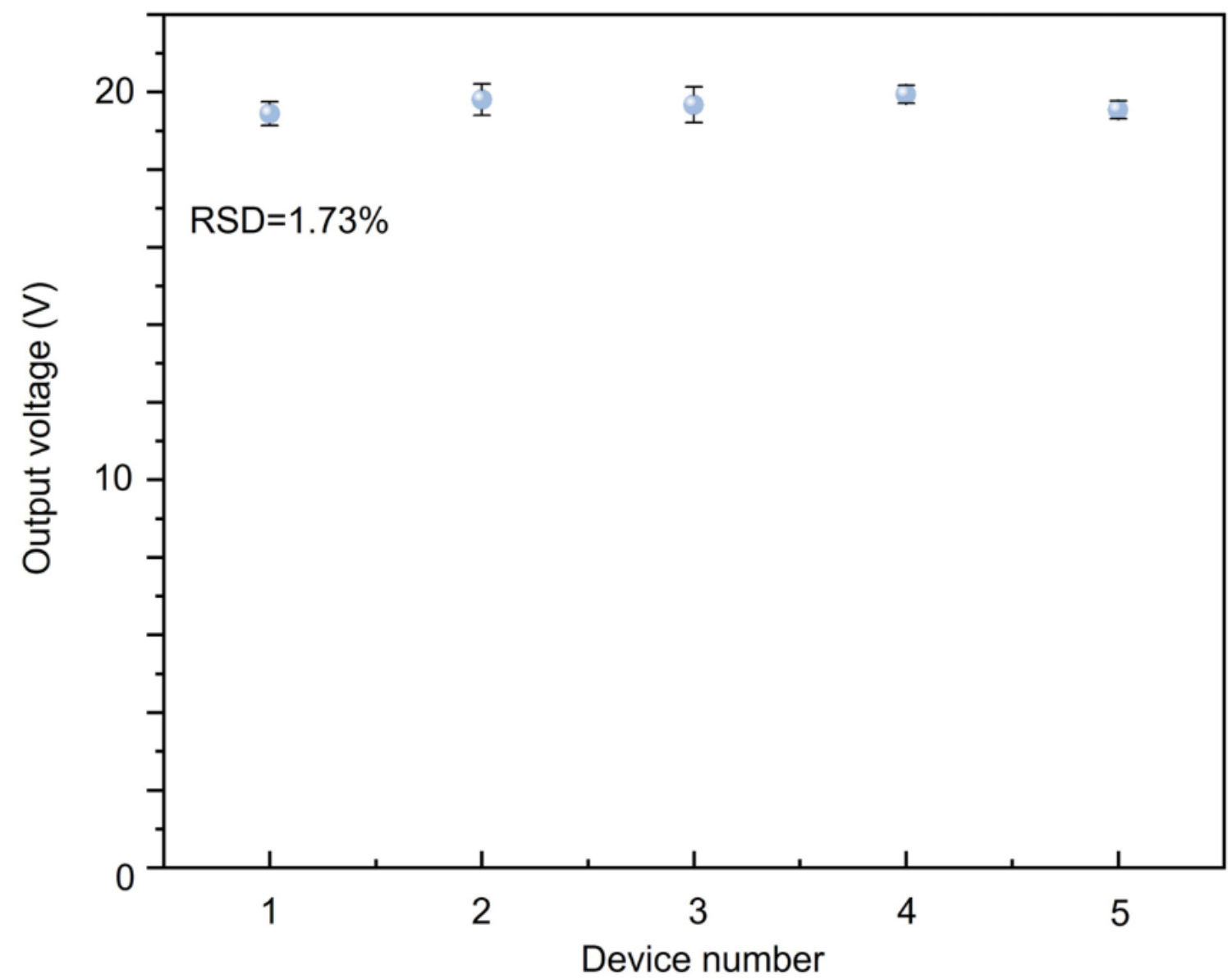

Figure S22. Repeatability of the manually driven device. Deionized water was used as the test liquid during the measurements.

Table S1. Comparison of the detection performance of the developed self-powered device with existing portable detection devices or methods.

Target substance	Method	Detection limit	Linear range	Ref.
ATP	ATP sensing paper assay	10^{-14} M	-	39
ATP	DNA tile response system	189.23 nM	0–2000 µM	40
ATP	Electrochemical sensor	41 nM	0.1 µM to 200 µM	41
ATP	Allosteric DNAzyme-based biosensor	2.2 pM	1 nM to 1 µM	42
miR-21	Electrochemical sensor	1.0 pM	Dynamic range (1 pM–100 nM))	43
miR-21	Label-free colorimetric	3.2 nM	10 nM–0.98 mM	44
miR-21	Surface enhanced Raman scattering	8.4 fM	10 fM–100 pM + 100 pM–40 nM	45
miR-21	Luminescent Biochip	26.3×10^3 fM	0.1−1 pM	46
ATP	Self-powered sensor	269 pM	1 nM–75 nM	This work
miR-21	Self-powered sensor	328 pM	0.5 nM–100 nM	This work

Movie S1.

Output electrical signal variations of the device operating in double-electrode mode and single-electrode mode.

Movie S2.

Operational demonstration of the manually-driven device.

REFERENCES

1. C. Chen, S. Ding, J. Wang, Digital health for aging populations. *Nat. Med.* **29**, 1623–1630 (2023).

2. S. M. Park, D. D. Won, B. J. Lee, D. Escobedo, A. Esteva, A. Aalipour, T. J. Ge, J. H. Kim, S. Suh, E. H. Choi, A. X. Lozano, C. Yao, S. Bodapati, F. B. Achterberg, J. Kim, H. Park, Y. Choi, W. J. Kim, J. H. Yu, A. M. Bhatt, J. K. Lee, R. Spitler, S. X. Wang, S. S. Gambhir, A mountable toilet system for personalized health monitoring via the analysis of excreta. *Nat. Biomed. Eng.* **4**, 624–635 (2020).

3. S. Tasoglu, Toilet-based continuous health monitoring using urine. *Nat. Rev. Urol.* **19**, 219–230 (2022).

4. Q. Zhong, E. K. Tan, C. Martin-Alonso, T. Parisi, L. Hao, J. D. Kirkpatrick, T. Fadel, H. E. Fleming, T. Jacks, S. N. Bhatia, Inhalable point-of-care urinary diagnostic platform. *Sci. Adv.* **10**, eadj9591 (2024).

5. X. He, T. Xu, Z. Gu, W. Gao, L. P. Xu, T. Pan, X. Zhang, Flexible and superwettable bands as a platform toward sweat sampling and sensing. *Anal. Chem.* **91**, 4296–4300 (2019).

6. U. M. Jalal, G. J. Jin, J. S. Shim, Paper-plastic hybrid microfluidic device for smartphone-based colorimetric analysis of urine. *Anal. Chem.* **89**, 13160–13166 (2017).

7. C. Yang, B. Wang, J. Wan, A. Kamath, F. You, B. Tian, Quantifying the global eco-footprint of wearable healthcare electronics. *Nature* **649**, 73–82 (2026).

8. H. Kim, S. Park, I. G. Jeong, S. H. Song, Y. Jeong, C. S. Kim, K. H. Lee, Noninvasive precision screening of prostate cancer by urinary multimarker sensor and artificial intelligence analysis. *ACS Nano* **15**, 4054–4065 (2021).

9. Y. Yang, J. Wang, W. Huang, G. Wan, M. Xia, D. Chen, Y. Zhang, Y. Wang, F. Guo, J. Tan, H. Liang, B. Du, L. Yu, W. Tan, X. Duan, Q. Yuan, Integrated urinalysis devices based on

interface-engineered field-effect transistor biosensors incorporated with electronic circuits. *Adv. Mater.* **34**, e2203224 (2022).

10. K. Stenberg, O. Hanssen, T. Edejer, M. Bertram, C. Brindley, A. Meshreky, J. Rosen, J. Stover, P. Verboom, R. Sanders, A. Soucat, Financing transformative health systems towards achievement of the health Sustainable Development Goals: a model for projected resource needs in 67 low-income and middle-income countries. *Lancet Glob. Health* **5**, e875–e887 (2017).

11. Y. Song, J. Min, Y. Yu, H. Wang, Y. Yang, H. Zhang, W. Gao, Wireless battery-free wearable sweat sensor powered by human motion. *Sci. Adv.* **6**, eaay9842 (2020).

12. Z. Wen, M.-H. Yeh, H. Guo, J. Wang, Y. Zi, W. Xu, J. Deng, L. Zhu, X. Wang, C. Hu, L. Zhu, X. Sun, Z. L. Wang, Self-powered textile for wearable electronics by hybridizing fiber-shaped nanogenerators, solar cells, and supercapacitors. *Sci. Adv.* **2**, e1600097 (2016).

13. Q. Zhou, J. Pan, S. Deng, F. Xia, T. Kim, Triboelectric nanogenerator-based sensor systems for chemical or biological detection. *Adv. Mater.* **33**, e2008276 (2021).

14. A. Gao, Q. Zhou, Z. Cao, W. Xu, K. Zhou, B. Wang, J. Pan, C. Pan, F. Xia, A self-powered biochemical sensor for intelligent agriculture enabled by signal enhanced triboelectric nanogenerator. *Adv. Sci.* **11**, e2309824 (2024).

15. X. Wei, B. Wang, X. Cao, H. Zhou, Z. Wu, Z. L. Wang, Dual-sensory fusion self-powered triboelectric taste-sensing system towards effective and low-cost liquid identification. *Nat. Food* **4**, 721–732 (2023).

16. J. Zhang, X. Wang, L. Zhang, S. Lin, S. Ciampi, Z. L. Wang, Triboelectric spectroscopy for in situ chemical analysis of liquids. *J. Am. Chem. Soc.* **146**, 6125–6133 (2024).

17. T. Zhao, Y. Fu, C. Sun, X. Zhao, C. Jiao, A. Du, Q. Wang, Y. Mao, B. Liu, Wearable biosensors for real-time sweat analysis and body motion capture based on stretchable fiber-based triboelectric nanogenerators. *Biosens. Bioelectron.* **205**, 114115 (2022).

18. P. Kanokpaka, L.-Y. Chang, B.-C. Wang, T.-H. Huang, M.-J. Shih, W.-S. Hung, J.-Y. Lai, K.-C. Ho, M.-H. Yeh, Self-powered molecular imprinted polymers-based triboelectric sensor for noninvasive monitoring lactate levels in human sweat. *Nano Energy* **100**, 107464 (2022).

19. P. Kanokpaka, Y.-H. Chang, C.-C. Chang, M. Rinawati, P.-C. Wang, L.-Y. Chang, M.-H. Yeh, Enabling glucose adaptive self-healing hydrogel based triboelectric biosensor for tracking a human perspiration. *Nano Energy* **112**, 108513 (2023).

20. X. Li, Y. Wei, Z. L. Wang, D. Wei, Scavenging energy and information through dynamically regulating the electrical double layer. *Adv. Funct. Mater.* **34**, 2405520 (2024).

21. S. Lin, X. Chen, Z. L. Wang, Contact electrification at the liquid-solid interface. *Chem. Rev.* **122**, 5209–5232 (2022).

22. W. H. Kim, J. U. Lee, M. J. Jeon, K. H. Park, S. J. Sim, Three-dimensional hierarchical plasmonic nano-architecture based label-free surface-enhanced Raman spectroscopy detection of urinary exosomal miRNA for clinical diagnosis of prostate cancer. *Biosens. Bioelectron.* **205**, 114116 (2022).

23. T. Antunes-Lopes, F. Cruz, Urinary biomarkers in overactive bladder: revisiting the evidence in 2019. *Eur. Urol. Focus* **5**, 329–336 (2019).

24. W. Xu, H. Zheng, Y. Liu, X. Zhou, C. Zhang, Y. Song, X. Deng, M. Leung, Z. Yang, R. X. Xu, Z. L. Wang, X. C. Zeng, Z. Wang, A droplet-based electricity generator with high instantaneous power density. *Nature* **578**, 392–396 (2020).

25. Q. Zhou, B. Wang, A. Gao, W. Xu, K. Zhou, J. Pan, G. Meng, C. Pan, F. Xia, Solution-tube-based volume eEffect triboelectric nanogenerator with Salt and pH Sensitivity. *Adv. Funct. Mater.* **32**, 2209100 (2022).

26. A. Zhu, S. Ali, Y. Xu, Q. Ouyang, Q. Chen, A SERS aptasensor based on AuNPs functionalized PDMS film for selective and sensitive detection of Staphylococcus aureus. *Biosens. Bioelectron.* **172**, 112806 (2021).

27. J. Pan, W. Xu, W. Li, S. Chen, Y. Dai, S. Yu, Q. Zhou, F. Xia, Electrochemical aptamer-based sensors with tunable detection range. *Anal. Chem.* **95**, 420–432 (2023).

28. Z. Hu, Y. Li, G. Figueroa-Miranda, S. Musall, H. Li, M. A. Martínez-Roque, Q. Hu, L. Feng, D. Mayer, A. Offenhäusser, Aptamer based biosensor platforms for neurotransmitters analysis. *Trends Anal. Chem.* **162**, 117021 (2023).

29. N. Nakatsuka, K.-A. Yang, J. M. Abendroth, K. M. Cheung, X. Xu, H. Yang, C. Zhao, B. Zhu, Y. S. Rim, Y. Yang, P. S. Weiss, M. N. Stojanović, A. M. Andrews, Aptamer-field-effect transistors overcome Debye length limitations for small-molecule sensing. *Science* **362**, 319–324 (2018).

30. S. C. Karunakaran, B. J. Cafferty, K. S. Jain, G. B. Schuster, N. V. Hud, Reversible transformation of a supramolecular hydrogel by redox switching of methylene blue-a noncovalent chain stopper. *ACS Omega* **5**, 344–349 (2020).

31. Q. He, Y. Han, Y. Huang, J. Gao, Y. Gao, L. Han, Y. Zhang, Reusable dual-enhancement SERS sensor based on graphene and hybrid nanostructures for ultrasensitive lead (II) detection. *Sens. Actuators B* **341**, 130031 (2021).

32. J. Liu, K. Jiang, L. Nguyen, Z. Li, T. Thundat, Interfacial friction-induced electronic excitation mechanism for tribo-tunneling current generation. *Mater. Horiz.* **6**, 1020–1026 (2019).

33. L. E. Helseth, Influence of salt concentration on charge transfer when a water front moves across a junction between a hydrophobic dielectric and a metal electrode. *Langmuir* **36**, 8002–8008 (2020).

34. F. Ricci, Y. Lai, J. Heeger, W. Plaxco, J. Sumner, Effect of molecular crowding on the response of an electrochemical DNA sensor. *Langmuir* **23**, 6827–6834 (2007).

35. S. Carlsson, N. Wiklund, L. Engstrand, E. Weitzberg, J. Lundberg, Effects of pH, Nitrite, and Ascorbic Acid on Nonenzymatic Nitric Oxide Generation and Bacterial Growth in Urine. *Nitric Oxide* **5**, 580–586 (2001).

36. Y. Wu, Y. He, J. Qi, S. Wang, Z. Wang, Urinary ATP may be a biomarker of interstitial cystitis/bladder pain syndrome and its severity. *Biomol. Biomed.* **24**, 170–175 (2024).

37. T. C. Chai, S. Keay, New theories in interstitial cystitis. *Nat. Clin. Pract. Urol.* **1**, 85–89 (2004).

38. N. Xue, S. Wu, Z. Li, X. Miao, Ultrasensitive and label-free detection of ATP by using gold nanorods coupled with enzyme assisted target recycling amplification. *Anal. Chim. Acta* **1104**, 117–124 (2020).

39. M. M. Calabretta, R. Álvarez-Diduk, E. Michelini, A. Roda, A. Merkoçi, Nano-lantern on paper for smartphone-based ATP detection. *Biosens. Bioelectron.* **150**, 111902 (2020).

40. D. Li, Y. Peng, L. Lu, D. Chen, B. Wang, Q. Pu, Q. Zhou, X. Xiao, M. Fu, X. Wang, J. Tu, H. Pei, Q. Wu, Programmed DNA tile response system enabled accurate detection of ATP for clinical diagnosis. *Chem. Eng. J.* **499**, 156270 (2024).

41. Z. Zhang, X. Zhao, J. Liu, J. Yin, X. Cao, Highly sensitive sandwich electrochemical sensor based on DNA-scaffolded bivalent split aptamer signal probe. *Sens. Actuators B* **311**, 127920 (2020).

42. Y. Shen, C. Shi, X. Ma, P. Hou, H. Zhang, J. Qin, L. Pan, G. Li, L. Gao, Q. Ma, D. Yang, M. Li, P. Wang, Allosteric DNAzyme-Enabled Sensitive and Multiplex Detection of Biomarkers for Rapid Diagnosis of Urinary Tract Infections. *Adv. Sci.* , e75417 (2026).

43. K. Boriachek, M. Umer, M. N. Islam, V. Gopalan, A. K. Lam, N.-T. Nguyen, M. J. A. Shiddiky, An amplification-free electrochemical detection of exosomal miRNA-21 in serum samples. *Analyst* **143**, 1662–1669 (2018).

44. H. Zhao, Y. Qu, F. Yuan, X. Quan, A visible and label-free colorimetric sensor for miRNA-21 detection based on peroxidase-like activity of graphene/gold-nanoparticle hybrids. *Anal. Methods* **8**, 2005–2012 (2016).

45. L. Fan, W. Liu, Q. Li, J. Lu, F. Jiang, Q. Yan, X. Liu, A Nanotechnology-Enhanced SERS Platform Integrated with Isothermal Amplification for Ultrasensitive Detection of Urinary miR-21 in Early Acute Kidney Injury Diagnosis. J. Wang, *J. Pharm. Anal.* **16**, 101585 (2026).

46. Y. Wang, Z. Li, Q. Lin, Y. Wei, J. Wang, Y. Li, R. Yang, Q. Yuan, Highly Sensitive Detection of Bladder Cancer-Related miRNA in Urine Using Time-Gated Luminescent Biochip. *ACS Sens.* **4**, 2124–2130 (2019).
